# The Validity and Reliability of Commercially Available Resistance Training Monitoring Devices: A Systematic Review

**DOI:** 10.1007/s40279-020-01382-w

**Published:** 2021-01-21

**Authors:** Jonathon Weakley, Matthew Morrison, Amador García-Ramos, Rich Johnston, Lachlan James, Michael H. Cole

**Affiliations:** 1 School of Behavioural and Health Sciences, Australian Catholic University, Building 211.1.26, Brisbane, QLD Australia; 2 Carnegie Applied Rugby Research (CARR) Centre, Institute of Sport, Physical Activity and Leisure, Leeds Beckett University, Leeds, UK; 3 Department of Sports Sciences and Physical Conditioning, Universidad Católica de la Santísima Concepción, Concepción, Chile; 4 Department of Physical Education and Sport, University of Granada, Granada, Spain; 5 Sport and Exercise Science, School of Allied Health, Human Services and Sport, La Trobe University, Melbourne, Australia

## Abstract

**Background:**

Monitoring resistance training has a range of unique difficulties due to differences in physical characteristics and capacity between athletes, and the indoor environment in which it often occurs. Traditionally, methods such as volume load have been used, but these have inherent flaws. In recent times, numerous portable and affordable devices have been made available that purport to accurately and reliably measure kinetic and kinematic outputs, potentially offering practitioners a means of measuring resistance training loads with confidence. However, a thorough and systematic review of the literature describing the reliability and validity of these devices has yet to be undertaken, which may lead to uncertainty from practitioners on the utility of these devices.

**Objective:**

A systematic review of studies that investigate the validity and/or reliability of commercially available devices that quantify kinetic and kinematic outputs during resistance training.

**Methods:**

Following PRISMA guidelines, a systematic search of SPORTDiscus, Web of Science, and Medline was performed; studies included were (1) original research investigations; (2) full-text articles written in English; (3) published in a peer-reviewed academic journal; and (4) assessed the validity and/or reliability of commercially available portable devices that quantify resistance training exercises.

**Results:**

A total of 129 studies were retrieved, of which 47 were duplicates. The titles and abstracts of 82 studies were screened and the full text of 40 manuscripts were assessed. A total of 31 studies met the inclusion criteria. Additional 13 studies, identified via reference list assessment, were included. Therefore, a total of 44 studies were included in this review.

**Conclusion:**

Most of the studies within this review did not utilise a gold-standard criterion measure when assessing validity. This has likely led to under or overreporting of error for certain devices. Furthermore, studies that have quantified intra-device reliability have often failed to distinguish between technological and biological variability which has likely altered the true precision of each device. However, it appears linear transducers which have greater accuracy and reliability compared to other forms of device. Future research should endeavour to utilise gold-standard criterion measures across a broader range of exercises (including weightlifting movements) and relative loads.

**Electronic supplementary material:**

The online version of this article (10.1007/s40279-020-01382-w) contains supplementary material, which is available to authorized users.

## Key Points

**Table:** 

For the accurate measurement of kinetic and kinematic outputs during resistance training, it is advised that linear transducers are utilised. These devices have demonstrated greater accuracy and reproducibility when compared to other technology.
It is strongly advised that future validity studies utilise gold-standard criterion measures across a range of relative intensities and exercises.
For the assessment of reliability, technological and biological error must be acknowledged and separated, so that the precision of each device during exercise can be accurately reported.

## Introduction

Resistance training is commonly used to improve strength, power, and lean body mass [1, 2], and is a fundamental part of athlete physical preparation. Traditionally, methods such as the number of repetitions or overall volume load (i.e., the multiplication of external mass, the number of repetitions and sets) have been used to quantify training loads [3–5]. However, these methods have fundamental errors that can reduce their application. For example, if an athlete utilises maximal intent or a pacing strategy, internal fatigue and adaptive responses can vastly differ [6, 7]. Furthermore, differences in exercise prescription, athlete physical capacity, and range of motion mean simple volume load equations can be misleading. This can be observed when completing differing repetition and set structures (e.g., three sets of 10 repetitions *vs.* 10 sets of three repetitions with the same external load) or when stronger athletes are compared against weaker counterparts [3, 4]. To circumvent these issues and support the accurate quantification of resistance training loads, a range of tools that assess kinetic and kinematic outputs have been developed [8–11]. By monitoring kinetic and kinematic outputs, changes in fatigue and proximity to concentric muscle failure can be closely monitored [6, 12, 13]. Furthermore, these devices have been used for a number of training purposes ranging from the immediate feedback of velocity and power outputs [14–17], to supporting full autoregulatory prescriptive methods [18, 19].

Linear position transducers (LPTs) and accelerometers are two commonly utilised tools that support the monitoring of training loads during resistance training [13, 20, 21]. While LPTs directly measure displacement and time, accelerometers are used to estimate kinetic and kinematic outputs by determining the time integral of the acceleration data. With respect to LPTs and accelerometers, there is an array of different brands, and these have been found to demonstrate varying levels of accuracy and reproducibility [9, 10]. It should be noted that LPTs should not be confused with linear velocity transducers (LVTs), which determine kinetic and kinematic outputs through the direct measurement of instantaneous velocity. Furthermore, in recent times, there have been a range of new devices that monitor resistance training outputs, with these being made possible through advancements in technology [22]. Examples of these include optic laser devices and the cameras within smartphones [22, 23]. While validity and reliability data have been published on these new devices, they have sparingly been compared to linear transducer (i.e., either LPTs or LVTs) and accelerometer data [24]. Furthermore, the literature has not been synthesised to inform practical use and help guide future research.

To support the accurate quantification of training loads, it is important that the technology used is both valid and reliable. This is particularly important for practitioners who utilise this information to make decisions regarding subsequent training sessions. The validity of an instrument often refers to its ability to measure what it is intended to measure with accuracy and precision [25–27]. This is typically quantified by comparing the output of the respective instrument to a ‘gold-standard’ or criterion measure. An example of a gold-standard measure would be the use of 3D high-speed motion capture when assessing velocity. Typical measures of validity include systematic and random bias, coefficient of variation (CV), and standard error of the estimate (SEE) [1, 28, 29]. Due to many resistance training methods now applying velocity loss thresholds with an aim to help mitigate fatigue responses [30, 31], or making programming decisions based on the force–velocity–power characteristics of an exercise [32], it is essential that outputs being produced are accurate. Otherwise, this may lead athletes to complete inappropriate training volumes or select exercises which may induce undue fatigue or generate a sub-optimal training stimulus.

The reliability of an instrument denotes its ability to reproduce measures on separate occasions when it is known that the measure of interest should not fluctuate [33]. When assessing devices that measure kinetic and kinematic outputs, both ‘intra-device’ (i.e., comparing outputs from the same device) and ‘inter-device’ (i.e., comparing outputs from two devices of the same make during the same trial) reliability are important. Intra-device reliability is essential to consider when tracking and identifying ‘meaningful’ changes over a specified period [34]. However, when assessing the reliability of an instrument, it is important to separate biological (i.e., human) and technological variation [22]. This is particularly pertinent during resistance training, where fluctuations in strength and readiness to train can cause substantial alterations in velocity and power outputs despite the same relative load being used [31, 35]. Therefore, research assessing reliability of devices needs to account for, and preferably remove, biological variation to gain a true insight into a device’s reproducibility. Inter-device reliability is important to consider when several devices of the same brand are being used in practice (e.g., two devices are being used to monitor two separate barbells when multiple athletes are training) [36, 37]. To ensure a true representation of each athlete’s capacity, the reproducibility of each device needs to be considered. Typical measures of reliability include typical/standard error of measurement (TEM/SEM), CV, and intra-class correlations (ICC) [25, 36, 38].

While there is an abundance of research that assesses the kinetic and kinematic outputs of commercially available devices during resistance training [1, 39, 40], there has not been a review assessing the validity and reliability of these different forms of technology. Due to the growing use of this equipment during resistance training, it is appropriate that a systematic review is completed to guide practitioners and researchers. Therefore, the aim of this review is to establish the level of evidence for: (1) the validity of all commercially available portable resistance training devices that monitor force, velocity, and power outputs; and, (2) the intra-device and inter-device reliability of these devices.

## Methods

### Search Strategy

Following PRISMA guidelines for systematic reviews [41], the academic databases SPORTDiscus, Web of Science, and Medline were systematically searched in August 2020 using titles, abstracts, keywords, and Boolean operators (AND/OR) to identify English-language peer-reviewed original research studies that investigated the validity and/or reliability of commercially available, portable devices that quantify kinetic and/or kinematic variables during resistance training. Studies were identified by searching abstracts, titles, and keywords for pre-determined terms relevant to the scope of this review (Table 1). All search results were extracted and imported into a reference manager (EndNote X9, Thomson Reuters, Philadelphia, PA, USA).

**Table 1:** Search terms and keywords utilised in each database search. Searches 1, 2 and 3 were combined using ‘AND’

Search 1	Search 2	Search 3	Search 4
“Linear position transducer” OR “Accelerometer” OR “High-speed camera” OR “Laser optics” OR “GymAware” OR “Push Band” OR “FLEX” OR “Tendo” OR “Beast sensor” OR “Trio-OptiTrack” OR “T-Force” OR “Chronojump” OR “Speed4Lift” OR “Velowin” OR “PowerLift” OR “WIMU” OR “iLOAD”	“Validity” OR “Reliability”	“Kinetic” OR “Kinematic” OR “Force” OR “Power” OR “Velocity”	“Strength training” OR “Resistance training” OR “Plyometrics”

### Selection Criteria

All duplicate studies were removed, and the titles and abstracts of all remaining studies were scanned for relevance by two authors (JW and MM). Studies that were deemed beyond the scope of the review were removed. The full text of the remaining studies was then assessed for eligibility. To be eligible for inclusion, studies were required to be (1) original research investigations; (2) full-text articles written in English; (3) published in a peer-reviewed academic journal before the 3rd of August, 2020; and (4) concerned with the validity and/or reliability of commercially available, portable, resistance training devices that monitor force, velocity, and power outputs during resistance training (i.e., exercise that consisted of applying an external load to the participant). If it was deemed that a study did not meet the inclusion criteria, it was excluded from the analysis. Additionally, if the study concerned a device that was no longer commercially available, it was not included. The reference lists of all eligible studies were then manually searched for any studies that were not retrieved in the initial search. If any studies were identified through this manual search strategy, it was subjected to the same assessment as previously described. Where necessary, means and measures of dispersion were extracted from figures in the manuscripts using WebPlotDigitizer v4.0 [42].

### Assessment of Reporting Quality

The reporting quality of the research was assessed using a modified version of the Downs and Black checklist [43] (Table 2). This method is valid for assessing the methodological reporting quality of observational study designs and has previously been utilised by systematic reviews pertaining to sport science [44]. Not all of the assessment criteria were applicable to the studies used in this review; thus, only 9 of the 27 criteria were used. These questions can be found in Electronic Supplementary Material Table S1. Study reporting quality was scored on a scale from ‘0’ (unable to determine, or no) to ‘1’ (yes). In total, a score of ‘9’ was indicative of the highest reporting quality, with scores above 6 being considered ‘good’, scores of 4–6 considered ‘moderate’, and scores below 4 being considered ‘poor’ methodological reporting quality.

**Table 2:** Methodological reporting quality of eligible studies used in systematic review

Study	Items assessed using modified Downs and Black checklist									
	Reporting						Internal validity			
	1	2	3	6	7	10	16	18	20	Total
Abbott et al. [59]	1	1	1	1	1	1	1	0	1	8
Askow et al. [25]	1	1	1	0	1	1	1	0	1	7
Balsalobre-Fernandez et al. [62]	1	1	1	1	1	1	1	0	1	8
Balsalobre-Fernandez et al. [69]	1	1	1	1	1	1	1	0	1	8
Balsalobre-Fernandez et al. [70]	1	1	1	1	1	1	1	0	1	8
Banyard et al. [8]	1	1	1	1	1	0	1	1	1	8
Beckham et al. [28]	1	1	0	1	1	0	1	1	1	7
Boehringer and Whyte [77]	1	1	0	1	1	1	1	0	1	7
Chéry and Ruf [60]	0	1	0	1	1	1	1	0	1	6
Comstock et al. [49]	0	1	0	0	0	0	0	0	1	2
Courel-Ibanez et al. [36]	1	1	1	1	0	1	1	1	1	8
Crewther et al. [50]	1	1	1	1	1	1	1	1	1	9
de Sa et al. [80]	1	1	1	1	1	1	1	1	1	9
Dorrell et al. [26]	1	1	1	1	1	1	1	1	1	9
Drinkwater et al. [27]	1	1	1	1	1	1	1	1	1	9
Fernandes et al. [55]	1	1	1	1	1	1	1	1	1	9
García-Mateo [63]	1	1	1	1	1	1	1	0	0	7
García-Pinillos et al. [61]	1	1	1	1	1	1	1	1	1	9
García-Ramos et al. [71]	1	1	1	1	1	1	1	1	1	9
Garnacho-Castano et al. [56]	1	1	0	1	1	1	1	1	1	8
Goldsmith et al. [53]	1	1	0	1	1	1	1	1	1	8
Gonzalez et al. [57]	1	1	0	1	1	1	1	1	1	8
Hughes et al. [52]	1	1	0	1	1	1	1	0	1	7
Jennings et al. [79]	1	1	0	1	1	1	1	1	1	8
Lake et al. [29]	1	1	1	1	1	1	1	1	1	9
Laza-Cagigas et al. [68]	1	1	1	1	1	1	1	1	1	9
Lorenzetti et al. [54]	1	1	1	1	0	0	1	0	1	6
Martinez-Cava et al. [51]	1	1	1	1	1	1	1	0	1	9
McGrath et al. [58]	1	1	0	1	1	0	1	0	1	7
Mitter et al. [9]	1	1	0	1	1	1	1	1	1	8
Muyor et al. [38]	1	1	1	1	1	1	1	1	1	9
Orange et al. [48]	1	1	1	1	1	1	1	1	1	9
Orange et al. [47]	1	1	1	1	1	1	1	1	1	9
Pino-Ortega et al. [66]	1	1	1	1	1	1	1	1	1	9
Peña García-Orea et al. [82]	1	1	1	1	1	0	1	0	1	9
Peña García-Orea et al. [72]	1	1	1	1	1	0	1	0	1	9
Perez-Castilla et al. [10]	1	1	1	1	1	1	1	1	1	9
Sanchez-Pay et al. [23]	1	1	1	1	1	1	1	1	1	9
Sanudo et al. [81]	1	1	1	1	1	1	1	1	1	9
Sato et al. [37]	1	1	0	1	1	1	1	0	1	7
Stock et al. [78]	1	1	0	1	0	1	1	1	1	7
Thompson et al. [24]	1	1	1	1	1	1	1	1	1	9
van den Tillaar and Ball [11]	1	1	1	1	1	1	1	0	1	8
Weakley et al. [22]	1	1	1	1	1	1	1	1	1	9

### Criteria for ‘Acceptable’ Validity and Reliability

Devices were deemed to have demonstrated ‘acceptable’ validity if the literature reported a very high correlation (> 0.70), moderate CV (< 10%), and a trivial or small ES (< 0.60) based on a modified effect size scale [45]. This is consistent with previous resistance training literature which has assessed the validity of resistance training devices [8, 22, 46]. Devices were said to demonstrate acceptable reliability if a device had an intra-class correlation coefficient ≥ 0.90, CV < 10%, and a standardised mean bias < 0.60. This is consistent with previous resistance training literature which has assessed the reliability of resistance training devices [22, 47, 48].

## Results

### Identification of Studies

**Fig. 1:**
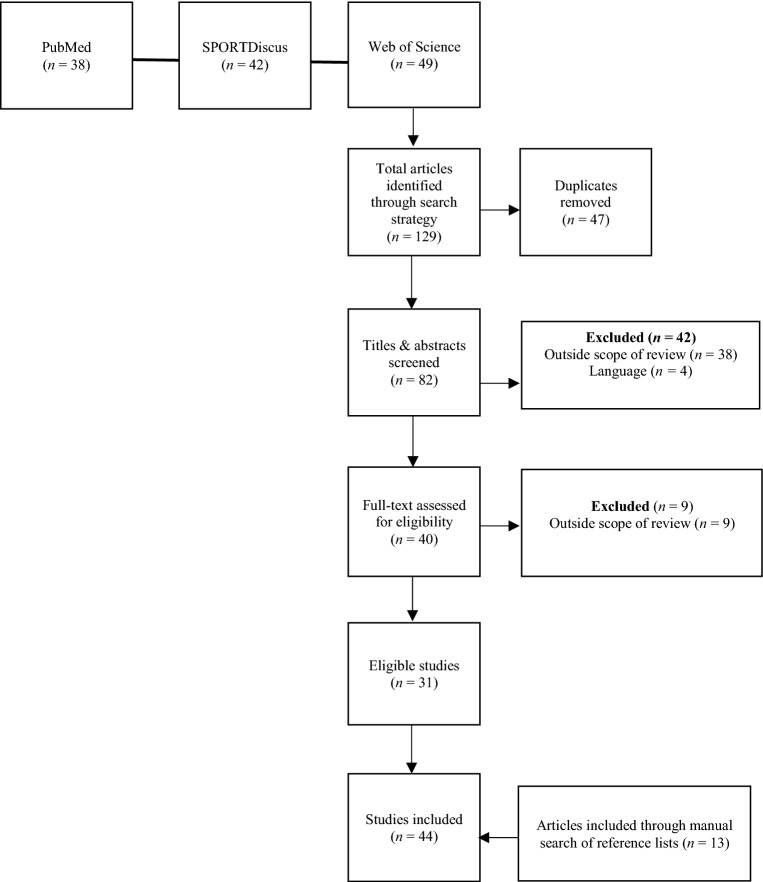
Flow of selection process of eligible studies for qualitative synthesis

The systematic search retrieved a total of 129 studies, with 47 of these being removed as duplicates. The titles and abstracts of the remaining 82 studies were screened, with 38 being deemed to be outside the scope of the review and a further 4 being excluded as they were not written in English. The full-text manuscripts of the remaining 40 studies were reviewed, resulting in the identification of 31 studies that met the inclusion criteria. The references lists of these 31 manuscripts were subsequently assessed, which led to an additional 13 studies being identified and a total of 44 studies included in this review. The identification process is outlined in Fig. 1.

### Research Reporting Quality

The methodological reporting quality of the research investigating the validity and/or reliability was relatively high [mean ± standard deviation 8.0 ± 1.3; median (interquartile range) 8 (1.25)] when appraised using the modified Downs and Black checklist [43] (Table 2). Items that were consistently not achieved included question 3 (full device details reported, *n* = 26 studies), 10 (actual statistics reported, *n* = 32 studies), and 18 (appropriate statistical analysis, *n* = 26 studies). To improve the quality of future research, authors should ensure that all statistics are reported and that the model and specifications of the device being used are included within the manuscript. Additionally, pooling of repeated measures must be accounted for with an appropriate statistical approach, while future research should seek to delineate the influence of technological variation from biological variation on reliability measures.

### Study Characteristics

All devices that were included within this review can be found within Table 3. Seven accelerometers (Push Band, Push Band 2.0, Beast Sensor, Bar Sensei, MyoTest, and Wimu System, RehaGait), 10 linear transducers [GymAware, SmartCoach, 1080Q, T-Force, Chronojump, Tendo, Speed4Lift, FitroDyne (Fitronic), Open Barbell System, and Musclelab (Ergotest)], three mobile applications (PowerLift/MyLift, iLoad, and Kinovea), and two optic devices (Velowin and Flex) were included. 36 studies assessed the validity (Tables 4, 5, 6, 7), while 28 studies investigated reliability (Tables 8, 9, 10, 11). The most common exercises assessed were the squat and bench press, either within the Smith machine or with free-weights, while velocity outputs were the most commonly assessed kinetic or kinematic variable.

**Table 3:** Summary of reliability and validity studies reported by device

Category	Device	Validity	Reliability
Accelerometer	PUSH Band	Balsalobre-Fernández et al. [62], Chery and Ruf [60], Courel-Ibanez et al. [36], McGrath et al. [58], Mitter et al. [9], Orange et al. [47], Perez-Castilla et al. [10], van den Tillar et al. [11], Sato et al. [37], Banyard et al. [8], Thompson et al. [24]	Balsalobre-Fernández et al. [52], Courel-Ibanez et al. [36], Orange et al. [47], Perez-Castilla et al. [10], van den Tillar et al. [11], Thompson et al. [24]
	PUSH Band 2.0	Hughes et al. [52], Lake et al. [29]	Hughes et al. [52], Lake et al. [29]
	Beast Sensor	Balsalobre-Fernández et al. [69], Mitter et al. [9], Perez-Castilla et al. [10], Thompson et al. [24]	Balsalobre-Fernández et al. [69], Perez-Castilla et al. [10], Thompson et al. [24]
	Bar Sensei	Beckham et al. [14], Thompson et al. [24], Abbott et al. [59]	Beckham et al. [14], Thompson et al. [24], Abbott et al. [59]
	MyoTest	Comstock et al. [49], Lorenzetti et al. [54], Crewther et al. [50]	Lorenzetti et al. [54]
	WIMU System	Garcia-Pinillos et al. [61], Muyor et al. [38], Pino-Ortega et al. [66]	Garcia-Pinillos et al. [61], Muyor et al. [38]
	RehaGait	García-Mateo [63]	García-Mateo [63]
Linear transducer	GymAware	Askow [25], Dorrell [26], Lorenzetti [54], Mitter [9], Crewther [50], Fernandes [55], Banyard [8], Drinkwater [27], Thompson et al. [24]	Askow et al. [25], Beckham et al. [28], Dorrell et al. [26], Hughes et al. [52], Lorenzetti et al. [54], Orange et al. [48], Thompson et al. [24]
	SmartCoach		Balsalobre-Fernández et al. [69]
	1080Q	Boehringer [77]	Boehringer et al. [77]
	T-Force	Lorenzetti [54], Perez-Castilla [10]	Courel-Ibanez et al. [3], Peña Garcia-Orea [72], Garcia-Pinillos et al. [61], Garcia-Ramos et al. [71], Lorenzetti et al. [54], Perez-Castilla et al. [10], Martinez-Cava et al. [51]
	Chronojump	Courel-Ibanez [36], Perez-Castilla [10]	Courel-Ibanez et al. [36], Perez-Castilla et al. [10]
	Tendo	Chery and Ruf [60], Garnacho-Castano et al. [56], Goldsmith et al. [53], Lorenzetti et al. [54], Gonzalez et al. [57], McGrath et al. [58]	Garnacho-Castano et al. [56], Lorenzetti et al. [54], Stock et al. [78]
	Speed4Lifts	Perez-Castilla et al. [10], Martinez-Cava et al. [51]	Perez-Castilla et al. [10], Martinez-Cava et al. [51]
	FitroDyne (fitronic)	Mitter [9], Fernandes [55]	Jennings et al. [79]
	Open Barbell System	Goldsmith [53], Gonzalez [57]	
	Musclelab (Ergotest)		van den Tillar et al. [11]
Mobile phone devices/applications	PowerLift	Courel-Ibanez [36], Balsalobre-Fernández [69], Balsalobre-Fernández [70], Perez-Castilla [7], Thompson et al. [24], Martinez-Cava et al. [51]	Balsalobre-Fernández et al. [69], Balsalobre-Fernández et al. [70], Courel-Ibanez et al. [36], Perez-Castilla et al. [10], Thompson et al. [24], Martinez-Cava et al. [51]
	iLoad v1.0	de Sa et al. [80]	
	Kinovea via Samsung S6	Sanchez-Pay et al. [23]	
	Kinovea via Xiaomi A1	Sanchez-Pay et al. [23]	
	Kinovea via iPhone X	Sanchez-Pay et al. [23]	
	Kinovea via Casio FH20	Sanchez-Pay et al. [23]	
	Kinovea via Digital Camera	Sanudo et al. [81]	
Optic devices	Velowin	Courel-Ibanez et al. [36], Peña Garcia-Orea et al. [82], Peña Garcia-Orea et al. [72], Garcia-Ramos et al. [71], Laza-Cagigas et al. [68], Perez-Castilla et al. [10]	Courel-Ibanez et al. [36], Peña Garcia-Orea et al. [82], Peña Garcia-Orea et al. [72], Garcia-Ramos et al. [71], Perez-Castilla et al. [10]
	Flex	Weakley et al. [22]	Weakley et al. [22]

**Table 4:** Summary of studies that investigated the validity of linear transducer devices used to measure kinetic and kinematic variables during resistance training

Study	Device	Criterion	Exercise	Intensity/load	Variable measured	Findings
Askow et al. [25]	GymAware	Qualysis Motion Capture System & AMTI Force Plate	F/W Back Squat	75–90%1RM	Mean velocity	ICC: 0.966; ES: 0.28
					Peak velocity	ICC: 0.982; ES: − 0.57
					Mean force	ICC: 0.992; ES: 0.11
					Peak force	ICC: 0.979; ES: − 0.03
					Mean power	ICC: 0.972; ES: 0.31
					Peak power	ICC: 0.993; ES: − 0.13
Banyard et al. [8]	GymAware	4 x Celesco PT5A-250 LPT & 1 x AMTI BP6001200 Force Plate	F/W Back Squat	20%1RM	Mean velocity	*r:* 0.96; CV: 3.6%; ES: 0.17; SEE: 0.04 m·s^−1^
					Peak velocity	*r:* 0.94; CV: 4.1%; ES: − 0.03; SEE: 0.08 m·s^−1^
					Mean force	*r:* 0.99; CV: 2.2%; ES: 0.57; SEE: 22.57N
					Peak force	*r:* 0.96; CV: 5.5%; ES: 0.61; SEE: 114.2N
					Mean power	*r:* 0.98; CV: 4%; ES: 0.72; SEE: 49.7W
					Peak power	*r:* 0.98; CV: 4.9%; ES: 0.81; SEE: 114.6W
				40%1RM	Mean velocity	*r:* 0.97; CV: 3.2%; ES: 0.19; SEE: 0.03 m·s^−1^
					Peak velocity	*r:* 0.97; CV: 2.9%; ES: 0.02; SEE: 0.05 m·s^−1^
					Mean force	*r:* 0.99; CV: 1.8%; ES: 0.37; SEE: 25.75N
					Peak force	*r:* 0.99; CV: 3.4%; ES: 0.38; SEE: 65.1N
					Mean power	*r:* 0.97; CV: 4.3%; ES: 0.6; SEE: 55.39W
					Peak power	*r:* 0.97; CV: 5.7%; ES: 0.67; SEE: 143.87W
				60%1RM	Mean velocity	*r:* 0.95; CV: 3.1%; ES: 0.11; SEE: 0.03 m·s^−1^
					Peak velocity	*r:* 0.96; CV: 3.7%; ES: − 0.02; SEE: 0.05 m·s^−1^
					Mean force	*r:* 0.99; CV: 1.7%; ES: 0.29; SEE: 29.33N
					Peak force	*r:* 0.99; CV: 3.3%; ES: 0.29; SEE: 74.63N
					Mean power	*r:* 0.97; CV: 4.7%; ES: 0.34; SEE: 66.02W
					Peak power	*r:* 0.97; CV: 5.7%; ES: 0.53; SEE: 152.84W
				80%1RM	Mean velocity	*r:* 0.96; CV: 3%; ES: 0.14; SEE: 0.02 m·s^−1^
					Peak velocity	*r:* 0.97; CV: 5.4%; ES: 0.07; SEE: 0.06 m·s^−1^
					Mean force	*r:* 0.99; CV: 1.6%; ES: 0.16; SEE: 34.88N
					Peak force	*r:* 0.99; CV: 3.5%; ES: 0.29; SEE: 79.78N
					Mean power	*r:* 0.99; CV: 3.5%; ES: 0.27; SEE: 37.79W
					Peak power	*r:* 0.98; CV: 6%; ES: 0.36; SEE: 145.79W
				90%1RM	Mean velocity	*r:* 0.96; CV: 2.8%; ES: 0.06; SEE: 0.02 m·s^−1^
					Peak velocity	*r:* 0.95; CV: 5.8%; ES: 0.12; SEE: 0.06 m·s^−1^
					Mean force	*r:* 1.00; CV: 1.1%; ES: 0.1; SEE: 23.83N
					Peak force	*r:* 0.99; CV: 3.2%; ES: 0.29; SEE: 85.27N
					Mean power	*r:* 0.99; CV: 4.7%; ES: 0.13; SEE: 44.9W
					Peak power	*r:* 0.96; CV: 7.7%; ES: 0.33; SEE: 166.75W
				100%1RM	Mean velocity	*r:* 0.99; CV: 2.5%; ES: 0.39; SEE: 0.01 m·s^−1^
					Peak velocity	*r:* 0.96; CV: 5.4%; ES: 0.04; SEE: 0.04 m·s^−1^
					Mean force	*r:* 1.00; CV: 0.7%; ES: 0.06; SEE: 16.9N
					Peak force	*r:* 0.96; CV: 5.5%; ES: 0.13; SEE: 140.28N
					Mean power	*r:* 0.99; CV: 5.3%; ES: 0.26; SEE: 23.89W
					Peak power	*r:* 0.95; CV: 7.3%; ES: 0.19; SEE: 126.8W
Boehringer and White [77]	1080Q	GymAware	S/M Bench Press	40–80%1RM	Mean velocity	MD: 0.018 ± 0.024 m·s^−1^; *r:*1.00
					Mean force	MD: − 116.3 ± 20.5N; *r:*0.99
					Mean power	MD: − 93.8 ± 35.3W; *r:*0.97
					Peak velocity	MD: 0.033 ± 0.026 m·s^−1^; *r:*1.00
					Peak force	MD: 193.0 ± 126.2N; *r:*0.91
					Peak power	MD: − 59.2 ± 35.9W; *r:*0.98
Chery & Ruf [60]	Tendo	GymAware	F/W Deadlift	20%1RM	Mean velocity	CV: 1.72%; MD: 0.03 m·s^−1^
					Peak velocity	CV: 1.03%; MD: − 0.16 m·s^−1^
				40%1RM	Mean velocity	CV: 2.17%; MD: 0.01 m·s^−1^
					Peak velocity	CV: 1.47%; MD: − 0.11 m·s^−1^
				60%1RM	Mean velocity	CV: 2.65%; MD: 0.01 m·s^−1^
					Peak velocity	CV: 1.08%; MD: − 0.09 m·s^−1^
				80%1RM	Mean velocity	CV: 2.99%; MD: 0.06 m·s^−1^
					Peak velocity	CV: 1.37%; MD: − 0.06 m·s^−1^
				90%1RM	Mean velocity	CV: 3.50%; MD: 0.04 m·s^−1^
					Peak velocity	CV: 1.36%; MD: − 0.04 m·s^−1^
				100%1RM	Mean velocity	CV: 4.07%; MD: 0.03 m·s^−1^
					Peak velocity	CV: 3.76%; MD: − 0.03 m·s^−1^
				All Loads	Mean velocity	CV: 4.08%; MD: 0.03 m·s^−1^
					Peak velocity	CV: 1.56%; MD: − 0.08 m·s^−1^
Courel-Ibanez et al. [36]	Chronojump	T-Force	S/M Bench Press	20–80 kg	Mean velocity	SEM: 0.05 m·s^−1^; CV: 6.1%; ICC: 0.992
					Mean propulsive velocity	SEM: 0.06 m·s^−1^; CV: 6.8%; ICC: 0.997
					PEAK velocity	SEM: 0.04 m·s^−1^; CV: 2.8%; ICC: 0.998
			S/M Back Squat	20–80 kg	Mean velocity	SEM: 0.04 m·s^−1^; CV: 10.6%; ICC: 0.984
					Mean propulsive velocity	SEM: 0.04 m·s^−1^; CV: 4.7%; ICC: 0.986
					Peak velocity	SEM: 0.05 m·s^−1^; CV: 2.9%; ICC: 0.989
			S/M Prone Bench Pull	20–80 kg	Mean velocity	SEM: 0.04 m·s^−1^; CV: 3.9%; ICC: 0.993
					Mean propulsive velocity	SEM: 0.03 m·s^−1^; CV: 3.2%; ICC: 0.994
					Peak velocity	SEM: 0.05 m·s^−1^; CV: 2.8%; ICC: 0.997
Crewther et al. 2011 [50]	GymAware	Force plate	F/W Loaded Squat Jump	20 kg	Peak force	*r: *0.59; bias: 202 ± 579N
					Peak power	*r: *0.67; bias: 401 ± 879W
				40 kg	Peak force	*r: *0.83; bias: 108 ± 255N
					Peak power	*r: *0.82; bias: 178 ± 611W
				60 kg	Peak force	*r: *0.87; bias: 39 ± 255N
					Peak power	*r: *0.74; bias: 45 ± 748W
				80 kg	Peak force	*r: *0.87; bias: 57 ± 414N
					Peak power	*r: *0.62; bias: 198 ± 762W
Dorrell et al. [26]	GymAware	Rapture-E 3D cameras and	F/W Back Squat	80%1RM	Bar displacement	MD: − 0.009 ± 0.005m
		force plate			Peak velocity	MD: 0.005 ± 0.007 m·s^−1^
					Mean velocity	MD: 0.029 ± 0.010 m·s^−1^
			F/W Bench Press	80%1RM	Bar displacement	MD: − 0.009 ± 0.009m
					Peak velocity	MD: 0.002 ± 0.007 m·s^−1^
					Mean velocity	MD: 0.017 ± 0.016 m·s^−1^
			F/W Deadlift	80%1RM	Bar displacement	MD: − 0.016 ± 0.009m
					Peak velocity	MD: 0.004 ± 0.004 m·s^−1^
					Mean velocity	MD: 0.100 ± 0.037 m·s^−1^
Drinkwater et al. [27]	GymAware	Sony Digital Video Recorder DRC-TRV900E (High speed camera)	S/M Back Squat	1 rep @ 3RM	Mean power (ecc)	SEE: 3.6W; CV: 1.16%; *r:* 1.00
					Mean power (con)	SEE: 4.1W; CV: 1.08%; *r:* 1.00
					Peak power (ecc)	SEE: 11.2W; CV: 1.43%; *r:* 1.00
					Peak power (con)	SEE: 14.4W; CV: 2.16%; *r: *1.00
			S/M Bench Throw	40 kg	Mean power (con)	SEE: 10.8W; CV: 2.78%; *r:* 0.97
					Max power (con)	SEE: 14.0W; CV: 1.85%; *r:* 0.99
			F/W Bench Press	1 rep @ 3RM	Mean power (ecc)	SEE: 7.1W; CV: 2.27%; *r:* 0.99
					Mean power (con)	SEE: 3.7W; CV: 1.50%; *r:* 1.00
					Peak power (ecc)	SEE: 7.8W; CV: 1.33%; *r:* 1.00
					Peak power (con)	SEE: 13.2W; CV: 3.02%; *r:* 0.99
Fernandes et al. [55]	FitroDyne (fitronic)	GymAware	S/M Bench Press	20%1RM	Peak velocity	LoA: 11.2 + 25.9 cm·s^−1^; *r:* 0.86
					Mean velocity	LoA: − 4.8 + 13.6 cm·s^−1^; *r:* 0.92
				30%1RM	Peak velocity	LoA: 10.7 + 22.9 cm·s^−1^; *r:* 0.79
					Mean velocity	LoA: − 2.0 + 13.1 cm·s^−1^; *r:* 0.88
				40%1RM	Peak velocity	LoA: 10.7 + 12.1 cm·s^−1^; *r:* 0.92
					Mean velocity	LoA: − 0.9 + 9.3 cm·s^−1^; *r: *0.89
				50%1RM	Peak velocity	LoA: 10.1 + 4.5 cm·s^−1^; *r:* 0.98
					Mean velocity	LoA: 0.1 + 9.5 cm·s^−1^ ; *r:* 0.86
				60%1RM	Peak velocity	LoA: 7.7 + 3.8 cm·s^−1^; *r:* 0.99
					Mean velocity	LoA: 0.0 + 11.6 cm·s^−1^; *r:* 0.86
				70%1RM	Peak velocity	LoA: 7.5 + 9.6 cm·s^−1^; *r: *0.95
					Mean velocity	LoA: 0.2 + 7.4 cm·s^−1^; *r:* 0.93
				80%1RM	Peak velocity	LoA: 5.5 + 10.1 cm·s^−1^; *r:* 0.96
					Mean velocity	LoA: 1.0 + 6.8 cm·s^−1^; *r:* 0.94
			S/M Back Squat	20%1RM	Peak velocity	LoA: 12.0 + 8.8 cm·s^−1^; *r:* 1.00
					Mean velocity	LoA: 2.0 + 6.3 cm·s^−1^; *r: *0.98
				30%1RM	Peak velocity	LoA: 10.6 + 9.3 cm·s^−1^; *r: *0.99
					Mean velocity	LoA: 0.6 + 7.5 cm·s^−1^; *r: *0.97
				40%1RM	Peak velocity	LoA: 10.4 + 9.1 cm·s^−1^; *r: *0.99
					Mean velocity	LoA: 1.2 + 5.4 cm·s^−1^; *r: *0.98
				50%1RM	Peak velocity	LoA: 9.2 + 7.4 cm·s^−1^ m·s^−1^; *r: *0.99
					Mean velocity	LoA: 2.3 + 3.3 cm·s^−1^; *r: *0.99
				60%1RM	Peak velocity	LoA: 8.5 + 5.8 cm·s^−1^; *r: *1.00
					Mean velocity	LoA: 2.0 + 3.0 cm·s^−1^; *r: *0.99
				70%1RM	Peak velocity	LoA: 8.3 + 5.4 cm·s^−1^; *r: *1.00
					Mean velocity	LoA: 2.2 + 2.1 cm·s^−1^; *r: *1.00
				80%1RM	Peak velocity	LoA: 8.0 + 6.5 cm·s^−1^; *r: *1.00
					Mean velocity	LoA: 1.1 + 6.0 cm·s^−1^; *r: *0.94
			S/M Bent Over Row	20%1RM	Peak velocity	LoA: 14.6 + 25.0 cm·s^−1^; *r: *0.94
					Mean velocity	LoA: − 0.1 + 12.0 cm·s^−1^; *r: *0.96
				30%1RM	Peak velocity	LoA: 14.6 + 18.9 cm·s^−1^; *r: *0.93
					Mean velocity	LoA: − 0.8 + 7.3 cm·s^−1^; *r: *0.97
				40%1RM	Peak velocity	LoA: 13.6 + 12.0 cm·s^−1^; *r: *0.97
					Mean velocity	LoA: 0.4 + 11.6 cm·s^−1^; *r: *0.91
				50%1RM	Peak velocity	LoA: 10.6 + 6.8 cm·s^−1^; *r: *0.98
					Mean velocity	LoA: 0.6 + 4.9 cm·s^−1^; *r: *0.98
				60%1RM	Peak velocity	LoA: 7.3 + 18.2 cm·s^−1^; *r: *0.92
					Mean velocity	LoA: − 0.3 + 6.0 cm·s^−1^; *r: *0.97
				70%1RM	Peak velocity	LoA: 7.7 + 15.3 cm·s^−1^; *r: *0.91
					Mean velocity	LoA: − 0.2 + 4.2 cm·s^−1^; *r: *0.98
				80%1RM	Peak velocity	LoA: 7.3 + 14.9 cm·s^−1^; *r: *0.92
					Mean velocity	LoA: − 0.2 + 3.9 cm·s^−1^; *r: *0.98
Garnacho-Castano et al. [56]	Tendo	T-Force	S/M Back Squat	40–60 kg, 85%1RM	Mean velocity	Bias: 0.02 ± 0.07 m·s^−1^; ICC: 0.985
					Peak velocity	Bias: − 0.08 ± 0.13 m·s^−1^; ICC: 0.963
					Mean power	Bias: 0.8 ± 44.31W; ICC: 0.966
				40–60 kg, 85%1RM	Peak power	Bias: − 209.99 ± 153.92W; ICC: 0.853
	Tendo	T-Force	S/M Bench Press		Mean velocity	Bias: 0.01 ± 0.06 m·s^−1^; ICC: 0.989
					Peak velocity	Bias: -0.06 ± 0.10 m·s^−1^; ICC: 0.963
					Mean power	Bias: 5.29 ± 38.48W; ICC: 0.968
					Peak power	Bias: − 105.13 ± 109.76W; ICC: 0.905
Goldsmith et al. [53]	Tendo	OptoTrak	F/W Back Squat	70%1RM	Mean velocity	LoA (95% CI): − 0.006357 (− 0.06042 to 0.04771) m·s^−1^; ICC: 0.9364
					Peak concentric velocity	LoA (95% CI): 0.07569 (− 0.05406 to 0.2054) m·s^−1^; ICC: 0.9362
	Open Barbell System	OptoTrak	F/W Back Squat	70%1RM	Mean velocity	LoA (95% CI): − 0.01163 (− 0.06855 to 0.04528) m·s^−1^; ICC: 0.8696
					Peak concentric velocity	LoA (95% CI): 0.03986 (− 0.1016 to 0.1813) m·s^−1^; ICC: 0.8351
Gonzalez et al. [57]	Open Barbell System	Tendo	F/W Back Squat	Overall	Peak velocity	MD: 0.11 + 0.01 m·s^−1^
					Mean velocity	MD: 0.01 + 0.01 m·s^−1^
				30%1RM	Peak velocity	*r:* 0.95; Bias: 0.163 m·s^−1^
					Mean velocity	*r:* 0.27; Bias: 0.012 m·s^−1^
				50%1RM	Peak velocity	*r: *0.73; Bias: 0.125 m·s^−1^
					Mean velocity	*r: *0.35; Bias: 0.007 m·s^−1^
				70%1RM	Peak velocity	*r: *0.87; Bias: 0.086 m·s^−1^
					Mean velocity	*r: *0.03; Bias: 0.008 m·s^−1^
				90%1RM	Peak velocity	*r:* 0.58; Bias: 0.053 m·s^−1^
					Mean velocity	*r: *0.25; Bias: 0.009 m·s^−1^
			F/W Front Squat	Overall	Peak velocity	MD: 0.11 + 0.01 m·s^−1^
					Mean velocity	MD: 0.01 + 0.01 m·s^−1^
				30%1RM	Peak velocity	*r:* 0.86; Bias: 0.158 m·s^−1^
					Mean velocity	*r: *0.44; Bias: 0.010 m·s^−1^
				50%1RM	Peak velocity	*r: *0.76; Bias: 0.119 m·s^−1^
					Mean velocity	*r: *− 0.27; Bias: 0.012 m·s^−1^
				70%1RM	Peak velocity	*r: *0.84; Bias: 0.091 m·s^−1^
					Mean velocity	*r: *0.29; Bias: 0.013 m·s^−1^
				90%1RM	Peak velocity	*r:* 0.49; Bias: 0.055 m·s^−1^
					Mean velocity	*r: *0.60; Bias: 0.007 m·s^−1^
Lorenzetti et al. [54]	T-Force	Vicon	F/W Back Squat	70%1RM	Mean velocity	RMSE: 0.070 m·s^−1^; MD: 0.062 m·s^−1^
					Maximum velocity	RMSE: 0.151 m·s^−1^; MD: 0.199 m·s^−1^
					Time to maximum velocity	RMSE: 0.026s; MD: 0.010s
			F/W Ballistic Squat	25 kg	Mean velocity	RMSE: 0.167 m·s^−1^; MD: 0.102 m·s^−1^
					Maximum velocity	RMSE: 0.263 m·s^−1^; MD: 0.150 m·s^−1^
					Time to maximum velocity	RMSE: 0.045s; MD: − 0.007s
	Tendo	Vicon	F/W Back Squat	70%1RM	Mean velocity	RMSE: 0.046 m·s^−1^; MD: 0.020 m·s^−1^
					Maximum velocity	RMSE: 0.194 m·s^−1^; MD: 0.159 m·s^−1^
					Time to maximum velocity	RMSE: 0.041s; MD: 0.031s
			F/W Ballistic Squat	25 kg	Mean velocity	RMSE: 0.157 m·s^−1^; MD: − 0.083 m·s^−1^
					Maximum velocity	RMSE: 0.315 m·s^−1^; MD: 0.217 m·s^−1^
					Time to maximum velocity	RMSE: 0.064s; MD: 0.046s
	GymAware	Vicon	F/W Back Squat	70%1RM	Mean velocity	RMSE: 0.064 m·s^−1^; MD: 0.046 m·s^−1^
					Maximum velocity	RMSE: 0.163 m·s^−1^; MD: 0.128 m·s^−1^
					Time to maximum velocity	RMSE: 0.042s; MD: 0.037s
			F/W Ballistic Squat	25 kg	Mean velocity	RMSE: 0.160 m·s^−1^; MD: − 0.091 m·s^−1^
					Maximum velocity	RMSE: 0.304 m·s^−1^; MD: 0.187 m·s^−1^
					Time to maximum velocity	RMSE: 0.046s; MD: 0.024s
Martinez-Cava et al. [51]	Speed4Lifts	T-Force	S/M Back Squat	25–95 kg	Peak velocity	SEM: 0.02 m·s^−1^; CV: 1.60%; ICC: 0.997
					Mean propulsive velocity	SEM: 0.03 m·s^−1^; CV: 3.09%; ICC: 0.995 *r*: 0.9936; SEE: 0.032 m·s^−1^
			S/M Bench Press	25–95 kg	Peak velocity	SEM: 0.06 m·s^−1^; CV: 4.94%; ICC: 0.995
					Mean propulsive velocity	SEM: 0.02 m·s^−1^; CV: 2.72%; ICC: 0.999* r*: 0.9985; SEE: 0.024 m·s^−1^
McGrath et al. [58]	Tendo Fitrodyne	3D Motion Camera Eagle Motion Camera	F/W Bench Press	40%1RM	Mean velocity	ICC: 0.977
				80%1RM	Mean velocity	
				Combined Load	Mean velocity	
Mitter et al. [9]	GymAware	Vicon	F/W Back Squat	30–100%1RM	Peak velocity	SEE: 0.019 m·s^−1^; RMSE: 0.025 m·s^−1^
					Mean velocity	SEE: 0.024 m·s^−1^; RMSE: 0.035 m·s^−1^
			F/W Bench Press	30–100%1RM	Peak velocity	SEE: 0.014 m·s^−1^; RMSE: 0.017 m·s^−1^
					Mean velocity	SEE: 0.030 m·s^−1^; RMSE: 0.041 m·s^−1^
			F/W Deadlift	30–100%1RM	Peak velocity	SEE: 0.017 m·s^−1^; RMSE: 0.021 m·s^−1^
					Mean velocity	SEE: 0.029 m·s^−1^; RMSE: 0.029 m·s^−1^
	FitroDyne (Fitronic)	Vicon	F/W Back Squat	30–100%1RM	Peak velocity	SEE: 0.022 m·s^−1^; RMSE: 0.043 m·s^−1^
					Mean velocity	SEE: 0.073 m·s^−1^; RMSE: 0.104 m·s^−1^
			F/W Bench Press	30–100%1RM	Peak velocity	SEE: 0.019 m·s^−1^; RMSE: 0.022 m·s^−1^
					Mean velocity	SEE: 0.100 m·s^−1^; RMSE: 0.162 m·s^−1^
			F/W Deadlift	30–100%1RM	Peak velocity	SEE: 0.018 m·s^−1^; RMSE: 0.035 m·s^−1^
					Mean velocity	SEE: 0.084 m·s^−1^; RMSE: 0.182 m·s^−1^
Perez-Castilla et al. [10]	T-Force	OptiTrack	S/M Bench Press	55–85%1RM	Mean velocity	Bias: − 0.01 ± 0.03 m·s^−1^
	Chronojump	OptiTrack	S/M Bench Press	55–85%1RM	Mean velocity	Bias: − 0.03 ± 0.03 m·s^−1^
	Speed4Lift	OptiTrack	S/M Bench Press	55–85%1RM	Mean velocity	Bias: − 0.04 ± 0.02 m·s^−1^
Thompson et al. [24]	GymAware	12 Camera Raptor 3D Motion Capture	F/W Back Squat	40%1RM	Mean velocity	*R*^2^: 0.95; LoA: − 0.02 ± 0.05 m·s^−1^
					Peak velocity	*R*^2^: 0.97; LoA: 0.01 ± 0.07 m·s^−1^
				50%1RM	Mean velocity	*R*^2^: 0.95; LoA: − 0.01 ± 0.04 m·s^−1^
					Peak velocity	*R*^2^: 0.98; LoA: 0.01 ± 0.06 m·s^−1^
				60%1RM	Mean velocity	*R*^2^: 0.98; LoA: − 0.02 ± 0.02 m·s^−1^
					Peak velocity	*R*^2^: 0.99; LoA: 0.01 ± 0.04 m·s^−1^
				70%1RM	Mean velocity	*R*^2^: 0.97; LoA: − 0.01 ± 0.03 m·s^−1^
					Peak velocity	*R*^2^: 0.99; LoA: 0.01 ± 0.05 m·s^−1^
				80%1RM	Mean velocity	*R*^2^: 0.99; LoA: − 0.01 ± 0.02 m·s^−1^
					Peak velocity	*R*^2^: 0.99; LoA: 0.02 ± 0.05 m·s^−1^
				90%1RM	Mean velocity	*R*^2^: 0.99; LoA: − 0.01 ± 0.01 m·s^−1^
					Peak velocity	*R*^2^: 0.96; LoA: 0.02 ± 0.10 m·s^−1^
				100%1RM	Mean velocity	*R*^2^: 0.97; LoA: − 0.02 ± 0.04 m·s^−1^
					Peak velocity	*R*^2^: 0.97; LoA: 0.01 ± 0.08 m·s^−1^
				Full	Mean velocity	*R*^2^: 0.99; LoA: − 0.01 ± 0.03 m·s^−1^
					Peak velocity	*R*^2^: 0.99; LoA: 0.01 ± 0.06 m·s^−1^
			Power Clean	40%1RM	Mean velocity	*R*^2^: 0.93; LoA: − 0.03 ± 0.08 m·s^−1^
					Peak velocity	*R*^2^: 0.91; LoA: − 0.01 ± 0.14 m·s^−1^
				50%1RM	Mean velocity	*R*^2^: 0.95; LoA: − 0.03 ± 0.05 m·s^−1^
					Peak velocity	*R*^2^: 0.93; LoA: − 0.01 ± 0.12 m·s^−1^
				60%1RM	Mean velocity	*R*^2^: 0.95; LoA: − 0.03 ± 0.05 m·s^−1^
					Peak velocity	*R*^2^: 0.95; LoA: − 0.01 ± 0.10 m·s^−1^
				70%1RM	Mean velocity	*R*^2^: 0.78; LoA: − 0.02 ± 0.07 m·s^−1^
					Peak velocity	*R*^2^: 0.95; LoA: 0.00 ± 0.08 m·s^−1^
				80%1RM	Mean velocity	*R*^2^: 0.86; LoA: − 0.02 ± 0.05 m·s^−1^
					Peak velocity	*R*^2^: 0.91; LoA: 0.00 ± 0.10 m·s^−1^
				90%1RM	Mean velocity	*R*^2^: 0.42; LoA: 0.00 ± 0.11 m·s^−1^
					Peak velocity	*R*^2^: 0.86; LoA: 0.01 ± 0.11 m·s^−1^
				100%1RM	Mean velocity	*R*^2^: 0.64; LoA: − 0.01 ± 0.07 m·s^−1^
					Peak velocity	*R*^2^: 0.86; LoA: 0.08 ± 0.11 m·s^−1^
				Full	Mean velocity	*R*^2^: 0.94; LoA: − 0.02 ± 0.07 m·s^−1^
					Peak velocity	*R*^2^: 0.96; LoA: 0.00 ± 0.11 m·s^−1^

*1RM* one repetition maximum, *SEE* standard error of the estimate, *MV* mean concentric velocity, *T-Force* T-force linear velocity transducer, *GymAware* GymAware PowerTool, *CV* coefficient of variation, *RMSE* root mean square of the estimate, *TEE* typical error of the estimate, *S/M* Smith machine, *F/W* free weight, *MD* mean difference, *r* Pearson’s correlation coefficient, *ICC* intraclass correlation coefficient, *SEM* standard error of measurement, *CMJ* countermovement jump, *ES* effect size, *ROM* range of motion, *ECC* eccentric, *CON* concentric, *LoA* limits of agreement

**Table 5:** Summary of studies that investigated the validity of accelerometer devices used to measure kinetic and kinematic variables during resistance training

Study	Device	Criterion	Exercise	Intensity/load	Variable measured	Findings
Abbott et al. [59]	Bar Sensei	Vicon (Nexus 1.8.5)	F/W Back Squat	20%1RM	Peak velocity	SEE: 0.06 m·s^−1^
					Mean velocity	SEE: 0.05 m·s^−1^
					Mean propulsive velocity	SEE: 0.11 m·s^−1^
				30%1RM	Peak velocity	SEE: 0.05 m·s^−1^
					Mean velocity	SEE: 0.04 m·s^−1^
					Mean propulsive velocity	SEE: 0.08 m·s^−1^
				40%1RM	Peak velocity	SEE: 0.05 m·s^−1^
					Mean velocity	SEE: 0.05 m·s^−1^
					Mean propulsive velocity	SEE: 0.07 m·s^−1^
				50%1RM	Peak velocity	SEE: 0.06 m·s^−1^
					Mean velocity	SEE: 0.05 m·s^−1^
					Mean propulsive velocity	SEE: 0.10 m·s^−1^
				60%1RM	Peak velocity	SEE: 0.09 m·s^−1^
					Mean velocity	SEE: 0.04 m·s^−1^
					Mean propulsive velocity	SEE: 0.06 m·s^−1^
				70%1RM	Peak velocity	SEE: 0.14 m·s^−1^
					Mean velocity	SEE: 0.04 m·s^−1^
					Mean propulsive velocity	SEE: 0.07 m·s^−1^
				80%1RM	Peak velocity	SEE: 0.17 m·s^−1^
					Mean velocity	SEE: 0.05 m·s^−1^
					Mean propulsive velocity	SEE: 0.05 m·s^−1^
				90%1RM	Peak velocity	SEE: 0.19 m·s^−1^
					Mean velocity	SEE: 0.04 m·s^−1^
					Mean propulsive velocity	SEE: 0.04 m·s^−1^
				100%1RM	Peak velocity	SEE: 0.16 m·s^−1^
					Mean velocity	SEE: 0.04 m·s^−1^
					Mean propulsive velocity	SEE: 0.03 m·s^−1^
Balsalobre-Fernández et al. [62]	Push Band	T-Force	S/M Back Squat	20–70 kg	Peak velocity	*r: *0.91; SEE: 0.1 m·s^−1^; MD: − 0.07 ± 0.1 m·s^−1^
					Mean velocity	*r: *0.86; SEE: 0.08 m·s^−1^; MD: 0.11 ± 0.1 m·s^−1^
Balsalobre-Fernández et al. [69]	Beast Sensor (wrist)	SmartCoach Power Encoder	F/W Back Squat	50–95%1RM	Mean velocity	Bias: 0.03 ± 0.06 m·s^−1^; SEE: 0.06 m·s^−1^
			F/W Bench Press	50–95%1RM	Mean velocity	Bias: 0.009 ± 0.04 m·s^−1^; SEE: 0.04 m·s^−1^
			F/W Hip-Thrust	50–95%1RM	Mean velocity	Bias: 0.06 ± 0.07 m·s^−1^; SEE: 0.07 m·s^−1^
	Beast Sensor (barbell)	SmartCoach Power Encoder	F/W Back Squat	50–95%1RM	Mean velocity	Bias: − 0.003 ± 0.05 m·s^−1^; SEE: 0.05 m·s^−1^
			F/W Bench Press	50–95%1RM	Mean velocity	Bias: 0.04 ± 0.05 m·s^−1^; SEE: 0.05 m·s^−1^
			F/W Hip-Thrust	50–95%1RM	Mean velocity	Bias: 0.03 ± 0.05 m·s^−1^; SEE: 0.04 m·s^−1^
Banyard et al. [8]	Push Band	4 x Celesco PT5A-250 LPT & 1 x AMTI BP6001200 Force Plate	F/W Back Squat	20%1RM	Mean velocity	*r:* 0.86; CV: 6.9%; ES: 0.24; SEE: 0.08 m·s^−1^
					Peak velocity	*r:* 0.80; CV: 8.1%; ES: 0.03; SEE: 0.16 m·s^−1^
					Mean force	*r:* 0.91; CV: 6.1%; ES: 2.29; SEE: 63.9N
					Peak force	*r:* 0.98; CV: 4%; ES: 0.04; SEE: 79.75N
					Mean power	*r:* 0.89; CV: 9.6%; ES: 0.28; SEE: 122.22W
					Peak power	*r:* 0.87; CV: 11.9%; ES: − 0.29; SEE: 299.89W
				40%1RM	Mean velocity	*r:* 0.80; CV: 8.2%; ES: 0.18; SEE: 0.08 m·s^−1^
					Peak velocity	*r:* 0.76; CV: 9.6%; ES: − 0.16; SEE: 0.15 m·s^−1^
					Mean force	*r:* 0.96; CV: 5%; ES: 1.41; SEE: 70.29N*r:* 0.98; CV: 4.7%; ES: 0.04; SEE: 109.92N
					Peak force	
					Mean power	*r:* 0.82; CV: 10.9%; ES: − 0.35; SEE: 145.92W
					Peak power	*r:* 0.81; CV: 14.2%; ES: − 0.5; SEE: 364.36W
				60%1RM	Mean velocity	*r:* 0.76; CV: 8.4%; ES: 0.49; SEE: 0.06 m·s^−1^
					Peak velocity	*r:* 0.74; CV: 10%; ES: − 0.22; SEE: 0.13 m·s^−1^
					Mean force	*r:* 0.96; CV: 5%; ES: 1.2; SEE: 87.53N
					Peak force	*r:* 0.97; CV: 5%; ES: 0.09; SEE: 117.37N
					Mean power	*r:* 0.79; CV: 13.1%; ES: − 0.36; SEE: 163.11W
					Peak power	*r:* 0.79; CV: 15.8%; ES: − 0.33; SEE: 383.25W
				80%1RM	Mean velocity	*r:* 0.73; CV: 10.7%; ES: 0.54; SEE: 0.05 m·s^−1^
					Peak velocity	*r:* 0.68; CV: 13.2%; ES: − 0.29; SEE: 0.14 m·s^−1^
					Mean force	*r:* 0.97; CV: 4.9%; ES: 0.83; SEE: 102.78N
					Peak force	*r:* 0.98; CV: 4.2%; ES: 0.07; SEE: 103.68N
					Mean power	*r:* 0.78; CV: 14.2%; ES: − 0.09; SEE: 126.79W
					Peak power	*r:* 0.78; CV: 18.7%; ES: − 0.5; SEE: 402.16W
				90%1RM	Mean velocity	*r:* 0.65; CV: 11.8%; ES: 0.69; SEE: 0.04 m·s^−1^
					Peak velocity	*r:* 0.65; CV: 12.9%; ES: − 0.52; SEE: 0.12 m·s^−1^
					Mean force	*r:* 0.98; CV: 3.4%; ES: 0.65; SEE: 77.28N
					Peak force	*r:* 0.97; CV: 4.9%; ES: 0.13; SEE: 135.34N
					Mean power	*r:* 0.67; CV: 22.7%; ES: 0.35; SEE: 163.23W
					Peak power	*r:* 0.81; CV: 17.1%; ES: − 0.41; SEE: 346.51W
				100%1RM	Mean velocity	*r:* 0.33; CV: 27.2%; ES: 1.62; SEE: 0.05 m·s^−1^
					Peak velocity	*r:* 0.49; CV: 18.4%; ES: − 0.13; SEE: 0.13 m·s^−1^
					Mean force	*r:* 0.97; CV: 5%; ES: 0.52; SEE: 115.17N
					Peak force	*r:* 0.92; CV: 8.3%; ES: 0.26; SEE: 213.05N
					Mean power	*r:* 0.32; CV: 38.3%; ES: 1.23; SEE: 162.09W
					Peak power	*r:* 0.66; CV: 18.7%; ES: − 0.18; SEE: 319.13W
Beckham et al. [28]	Bar Sensei	GymAware	F/W Back Squat	45%1RM	Mean velocity	ICC: 0.482; MD: − 0.106 m·s^−1^
					Peak velocity	ICC: 0.555; MD: 0.009 m·s^−1^
				60%1RM	Mean velocity	ICC: 0.303; MD: − 0.094 m·s^−1^
					Peak velocity	ICC: 0.362; MD: − 0.037 m·s^−1^
				75%1RM	Mean velocity	ICC: 0.329; MD: − 0.081 m·s^−1^
					Peak velocity	ICC: 0.361; MD: − 0.099 m·s^−1^
Chery & Ruf [60]	PUSH Band	GymAware	F/W Deadlift	20%1RM	Mean velocity	CV: 11.21%; MD: 0.05 m·s^−1^CV: 10.63%; MD: 0.12 m·s^−1^
					Peak velocity	
				40%1RM	Mean velocity	CV: 17.51%; MD: − 0.10 m·s^−1^
					Peak velocity	CV: 15.23%; MD:− 0.16 m·s^−1^
				60%1RM	Mean velocity	CV: 9.51%; MD: 0.02 m·s^−1^
					Peak velocity	CV: 10.34%; MD: 0.02 m·s^−1^
				80%1RM	Mean velocity	CV: 16.02%; MD: 0.13 m·s^−1^CV: 11.76%; MD: 0.18 m·s^−1^
					Peak velocity	
				90%1RM	Mean velocity	CV: 14.23%; MD: 0.10 m·s^−1^
					Peak velocity	CV: 8.15%; MD: 0.12 m·s^−1^
				100%1RM	Mean velocity	CV: 35.00%; MD: 0.08 m·s^−1^
					Peak velocity	CV: 33.34%; MD: 0.13 m·s^−1^
				All Loads	Mean velocity	CV: 22.69%; MD: 0.05 m·s^−1^
					Peak velocity	CV: 20.35%; MD: 0.07 m·s^−1^
Comstock et al. [48]	Myotest	Ballistic Measurement System force plate and Celesco linear transducer	S/M Bench Press	100% 1RM	Peak force	*R*^*2*^:0.92
			S/M Bench Throw	30% 1RM	Peak power	*R*^*2*^: 0.92
			S/M Squat	100% 1RM	Peak force	*R*^*2*^: 0.97
			S/M Squat Jump	30% 1RM	Peak power	*R*^*2*^: 0.82
Courel-Ibanez et al. [36]	Push Band	T-Force	S/M Bench Press	20–80 kg	Mean velocity	SEM: 0.13 m·s^−1^; CV: 18.3%; ICC: 0.928
					Peak velocity	SEM: 0.23 m·s^−1^; CV: 17.1%; ICC: 0.937
			S/M Back Squat	20–80 kg	Mean velocity	SEM: 0.07 m·s^−1^; CV: 8.8%; ICC: 0.941
					Peak velocity	SEM: 0.10 m·s^−1^; CV: 6.4%; ICC: 0.952
Crewther et al. [50]	MyoTest	Force Plate	F/W Loaded squat jump	20 kg	Peak force	*r: *0.87; bias: 171 ± 336N
					Peak power	*r: *0.66; bias: 141 ± 896W
				40 kg	Peak force	*r: *0.89; bias: 73 ± 256N
					Peak power	*r: *0.88; bias: − 180 ± 593W
				60 kg	Peak force	*r: *0.95; bias: 32 ± 196N
					Peak power	*r: *0.82; bias: − 112 ± 610W
				80 kg	Peak force	*r: *0.97; bias: 7 ± 219N
					Peak power	*r: *0.90; bias: 23 ± 400W
Garcia-Pinillos et al. [61]	WIMU System	T-Force	S/M Concentric-Only Half ROM Back Squat	10–100%1RM	Mean velocity	Bias: 0.02 ± 0.06 m·s^−1^
					Mean propulsive velocity	Bias: 0.06 ± 0.07 m·s^−1^
					Maximum velocity	Bias: 0.16 ± 0.16 m·s^−1^
				10%1RM	Mean velocity	*r*: 0.865
					Mean propulsive velocity	*r*: 0.898
					Maximum velocity	*r*: 0.971
				20%1RM	Mean velocity	*r*: 0.520
					Mean propulsive velocity	*r*: 0.398
					Maximum velocity	*r*: 0.773
				30%1RM	Mean velocity	*r*: 0.696
					Mean propulsive velocity	*r*: 0.813
					Maximum velocity	*r*: 0.196
				40%1RM	Mean velocity	*r*: 0.877
					Mean propulsive velocity	*r*: 0.882
					Maximum velocity	*r*: 0.842
				50%1RM	Mean velocity	*r*: 0.760
					Mean propulsive velocity	*r*: 0.823
					Maximum velocity	*r*: 0.908
				60%1RM	Mean velocity	*r*: 0.646
					Mean propulsive velocity	*r*: 0.645
					Maximum velocity	*r*: 0.729
				70%1RM	Mean velocity	*r*: 0.419
					Mean propulsive velocity	*r*: 0.628
					Maximum velocity	*r*: 0.819
				80%1RM	Mean velocity	*r*: 0.662
					Mean propulsive velocity	*r*: 0.632
					Maximum velocity	*r*: 0.498
				90%1RM	Mean velocity	*r*: 0.739
					Mean propulsive velocity	*r*: 0.717
					Maximum velocity	*r*: 0.742
				100%1RM	Mean velocity	*r*: 0.687
					Mean propulsive velocity	*r*: 0.685
					Maximum velocity	*r*: 0.861
Hughes et al. [52]	PUSH Band 2.0 (body)	GymAware	F/W Back Squat	30–90%1RM	Mean velocity	*r: *0.99
			F/W Bench Press		Mean velocity	*r: *0.99
			F/W Bench Pull		Mean velocity	*r: *0.98
			F/W OH Press		Mean velocity	*r: *0.99
			S/M Back Squat	30–90%1RM	Mean velocity	*r: *0.97
			S/M Bench Press		Mean velocity	*r:* 0.99
			S/M Bench Pull		Mean velocity	*r:* 0.99
			S/M OH Press		Mean velocity	*r:* 0.99
	PUSH Band 2.0 (bar)	GymAware	F/W Back Squat	30–90%1RM	Mean velocity	*r*: 0.97
			F/W Bench Press		Mean velocity	*r*: 0.99
			F/W OH Press		Mean velocity	*r*: 0.96
			S/M Back Squat	30–90%1RM	Mean velocity	*r:* 0.97
			S/M Bench Press		Mean velocity	*r*: 0.98
			S/M OH Press		Mean velocity	*r*: 0.96
Lake et al. [29]	PUSH Band 2.0	Vicon T40S	F/W Bench Press	60%1RM	Peak velocity	MD: − 0.039 m·s^−1^; LPR: 0.907
					Mean velocity	MD: − 0.065 m·s^−1^; LPR: 0.797
			F/W Bench Press	90%1RM	Peak velocity	MD: − 0.063 m·s^−1^; LPR: 1.110
					Mean velocity	MD: − 0.038 m·s^−1^; LPR: 0.816
Lorenzetti et al. [54]	MyoTest	Vicon	F/W Ballistic Squat	25 kg	Mean velocity	RMSE: 0.233 m·s^−1^; MD: 0.149 m·s^−1^
					Maximum velocity	RMSE: 0.418 m·s^−1^; MD: 0.278 m·s^−1^
					Time to maximum velocity	RMSE: 0.054s; MD: − 0.034s
Garcia-Mateo [63]	RehaGait (bar)RehaGait (body)	MyLift via iPhone 6 iOS 9.3.2MyLift via iPhone 6 iOS 9.3.2	F/W Squat with arms extended	<1 kg	Mean velocity	MD ± SD: 0.364 ± 0.069 m·s^−1^
			F/W Squat with arms extended	<1 kg	Mean velocity	MD ± SD: 0.318 ± 0.08 m·s^−1^
McGrath et al. [58]	PUSH Band	3D Motion Camera Eagle Motion Camera	F/W Bench Press	40%1RM	Mean velocity	Mean ± SD: 0.746 ± 0.124 m·s^−1^; CV: 16.62%
				80%1RM	mean velocity	Mean ± SD: 0.322 ± 0.124 m·s^−1^; CV: 38.50%
				Combined Load	Mean velocity	Mean ± SD: 0.510 ± 0.244 m·s^−1^; CV: 47.83%*; *ICC: 0.923
Mitter et al. [9]	Beast Sensor	Vicon	F/W Back Squat	30–100%1RM	Peak velocity	SEE: 0.176 m·s^−1^; RMSE: 0.320 m·s^−1^SEE: 0.116 m·s^−1^; RMSE: 0.177 m·s^−1^
					Mean velocity	
			F/W Bench Press	30–100%1RM	Peak velocity	SEE: 0.113 m·s^−1^; RMSE: 0.134 m·s^−1^
					Mean velocity	SEE: 0.084 m·s^−1^; RMSE: 0.098 m·s^−1^
			F/W Deadlift	30–100%1RM	Peak velocity	SEE: 0.147 m·s^−1^; RMSE: 0.361 m·s^−1^
					Mean velocity	SEE: 0.105 m·s^−1^; RMSE: 0.200 m·s^−1^
	PUSH Band	Vicon	F/W Back Squat	30–100%1RM	Peak velocity	SEE: 0.137 m·s^−1^; RMSE: 0.229 m·s^−1^
					Mean velocity	SEE: 0.078 m·s^−1^; RMSE: 0.147 m·s^−1^
			F/W Bench Press	30–100%1RM	Peak velocity	SEE: 0.113 m·s^−1^; RMSE: 0.121 m·s^−1^
					Mean velocity	SEE: 0.065 m·s^−1^; RMSE: 0.101 m·s^−1^
			F/W Deadlift	30–100%1RM	Peak velocity	SEE: 0.183 m·s^−1^; RMSE: 0.235 m·s^−1^
					Mean velocity	SEE: 0.105 m·s^−1^; RMSE: 0.136 m·s^−1^
Muyor et al [38]	WIMU	T-Force	S/M Back Squat	40%1RM	Mean velocity (con)	ES: 0.34*d*; SEM: 0.003 m·s^−1^; ICC: 0.970
					Mean velocity (ecc)	ES: 0.21*d*; SEM: 0.007 m·s^−1^; ICC: 0.971
				80%1RM	Mean velocity (con)	ES: 0.25*d*; SEM: 0.003 m·s^−1^; ICC: 0.976
					Mean velocity (ecc)	ES: 0.44*d*; SEM: 0.005 m·s^−1^; ICC: 0.953
Orange et al. [47]	PUSH Band PUSH Band	GymAware	F/W Back Squat	20%1RM	Mean velocity	Standardised mean bias: 0.61 m·s^−1^; *r:*0.80
					Peak velocity	Standardised mean bias: 0.53 m·s^−1^;* r:*0.80
					Mean power	Standardised mean bias: 0.51W;* r:*0.91
					Peak power	Standardised mean bias: 1.20W;* r:*0.90
				40%1RM	Mean velocity	Standardised mean bias: 1.17 m·s^−1^;* r*: 0.72
					Peak velocity	Standardised mean bias: 1.20 m·s^−1^;* r:*0.70
					Mean power	Standardised mean bias: 1.10W;* r:*0.77
					Peak power	Standardised mean bias: 1.01W;* r:*0.88
				60%1RM	Mean velocity	Standardised mean bias: 1.41 m·s^−1^; *r: *0.78
					Peak velocity	Standardised mean bias: 1.58 m·s^−1^; *r:*0.68
					Mean power	Standardised mean bias: 1.73W;* r:*0.76
					Peak power	Standardised mean bias: 1.39W;* r:*0.77
				80%1RM	Mean velocity	Standardised mean bias: 2.23 m·s^−1^;* r: *0.79
					Peak velocity	Standardised mean bias: 2.23 m·s^−1^;* r:*0.84
					Mean power	Standardised mean bias: 2.24W;* r:*0.76
					Peak power	Standardised mean bias: 1.43W;* r:*0.85
				90%1RM	Mean velocity	Standardised mean bias: 2.61 m·s^−1^; *r:*0.66
					Peak velocity	Standardised mean bias: 2.74 m·s^−1^;* r:*0.41
					Mean power	Standardised mean bias: 2.08W;* r:*0.67
					Peak power	Standardised mean bias: 1.59W;* r:*0.59
		GymAware	F/W Bench Press	20%1RM	Mean velocity	Standardised mean bias: 1.06 m·s^−1^;* r: 0.30*
					Peak velocity	Standardised mean bias: 0.55 m·s^−1^;* r:*0.44
					Mean power	Standardised mean bias: 0.35W;* r:*0.50
					Peak power	Standardised mean bias: 0.74W;* r:*0.66
				40%1RM	Mean velocity	Standardised mean bias: 0.68 m·s^−1^;* r: *0.85
					Peak velocity	Standardised mean bias: 0.37 m·s^−1^;* r:*0.73
					Mean power	Standardised mean bias: 0.31W;* r:*0.88
					Peak power	Standardised mean bias: 0.73W;* r:*0.59
				60%1RM	Mean velocity	Standardised mean bias: 0.55 m·s^−1^;* r: *0.59
					Peak velocity	Standardised mean bias: 0.18 m·s^−1^;* r:*0.52
					Mean power	Standardised mean bias: 0.00W;* r:*0.68
					Peak power	Standardised mean bias: 0.70W;* r:*0.62
				80%1RM	Mean velocity	Standardised mean bias: 1.03 m·s^−1^;* r*: 0.73
					Peak velocity	Standardised mean bias: 0.00 m·s^−1^;* r: *0.50
					Mean power	Standardised mean bias: 0.64W;* r:*0.76
					Peak power	Standardised mean bias: 0.88W;* r:*0.46
				90%1RM	Mean velocity	Standardised mean bias: 1.12 m·s^−1^;* r*: 0.44
					Peak velocity	Standardised mean bias: 0.10 m·s^−1^;* r: *0.45
					Mean power	Standardised mean bias: 0.60W;* r:*0.48
					Peak power	Standardised mean bias: 0.48W;* r:*0.49
Perez-Castilla et al. [10]	PUSH Band	OptiTrack	S/M Bench Press	55–85%1RM	Mean velocity	Bias: 0.10 ± 0.06 m·s^−1^
	Beast sensor	OptiTrack	S/M Bench Press	55–85%1RM	Mean velocity	Bias: 0.05 ± 0.21 m·s^−1^
Pino-Ortega et al. [66]	WIMU	GymAware	Leg Extension Machine	30–90 kg	Mean linear velocity	*R*^*2*^:0.9995; Bias: 0.011 ± 0.006 m·s^−1^; LoA: − 0.024 to 0.01 m·s^−1^
Sato et al. [37]	PUSH Band	Vicon (Nexus 1.8.5)	DB Biceps Curl	4.54–6.82 kg	Mean velocity	TE: 0.060 m·s^−1^; RTE: 7.2%; *r*: 0.883
					Peak velocity	TE: 0.105 m·s^−1^; RTE: 6.5%; *r: *0.923
			DB Shoulder Press	4.54–6.82 kg	Mean velocity	TE: 0.090 m·s^−1^; RTE: 12.6%; *r: *0.864
					Peak velocity	TE: 0.163 m·s^−1^; RTE: 14.0%; *r: *0.801
Thompson et al. [24]	PUSH Band (body) PUSH Band (bar) Bar Sensei Beast Sensor	12 Camera raptor 3D motion capture	F/W Back Squat	40%1RM	Mean velocity	*R*^2^: 0.92; LoA: 0.00 ± 0.06 m·s^−1^
					Peak velocity	*R*^2^: 0.94; LoA: 0.09 ± 0.10 m·s^−1^
				50%1RM	Mean velocity	*R*^2^: 0.96; LoA: 0.00 ± 0.04 m·s^−1^
					Peak velocity	*R*^2^: 0.76; LoA: 0.09 ± 0.22 m·s^−1^
				60%1RM	Mean velocity	*R*^2^: 0.95; LoA: 0.01 ± 0.05 m·s^−1^
					Peak velocity	*R*^2^: 0.45; LoA: 0.12 ± 0.33 m·s^−1^
				70%1RM	Mean velocity	*R*^2^: 0.88; LoA: 0.02 ± 0.07 m·s^−1^
					Peak velocity	*R*^2^: 0.60; LoA: 0.11 ± 0.29 m·s^−1^
				80%1RM	Mean velocity	*R*^2^: 0.92; LoA: 0.00 ± 0.08 m·s^−1^
					Peak velocity	*R*^2^: 0.37; LoA: 0.13 ± 0.44 m·s^−1^
				90%1RM	Mean velocity	*R*^2^: 0.79; LoA: 0.00 ± 0.10 m·s^−1^
					Peak velocity	*R*^2^: 0.53; LoA: 0.15 ± 0.30 m·s^−1^
				100%1RM	Mean velocity	*R*^2^: 0.58; LoA: − 0.07 ± 0.10 m·s^−1^
					Peak velocity	*R*^2^: 0.48; LoA: 0.12 ± 0.35 m·s^−1^
				Full	Mean velocity	*R*^2^: 0.97; LoA: − 0.01 ± 0.09 m·s^−1^
					Peak velocity	*R*^2^: 0.80; LoA: 0.12 ± 0.30 m·s^−1^
			Power clean	40%1RM	Mean velocity	*R*^2^: 0.38; LoA: 0.07 ± 0.24 m·s^−1^
					Peak velocity	*R*^2^: 0.27; LoA: 0.61 ± 0.40 m·s^−1^
				50%1RM	Mean velocity	*R*^2^: 0.50; LoA: 0.07 ± 0.23 m·s^−1^
					Peak velocity	*R*^2^: 0.43; LoA: 0.60 ± 0.38 m·s^−1^
				60%1RM	Mean velocity	*R*^2^: 0.50; LoA: 0.07 ± 0.18 m·s^−1^
					Peak velocity	*R*^2^: 0.24; LoA: 0.56 ± 0.42 m·s^−1^
				70%1RM	Mean velocity	*R*^2^: 0.66; LoA: 0.07 ± 0.18 m·s^−1^
					Peak velocity	*R*^2^: 0.43; LoA: 0.59 ± 0.32 m·s^−1^
				80%1RM	Mean velocity	*R*^2^: 0.54; LoA: 0.07 ± 0.22 m·s^−1^
					Peak velocity	*R*^2^: 0.27; LoA: 0.58 ± 0.36 m·s^−1^
				90%1RM	Mean velocity	*R*^2^: 0.61; LoA: 0.10 ± 0.19 m·s^−1^
					Peak velocity	*R*^2^: 0.60; LoA: 0.62 ± 0.24 m·s^−1^
				100%1RM	Mean velocity	*R*^2^: 0.34; LoA: 0.09 ± 0.16 m·s^−1^
					Peak velocity	*R*^2^: 0.66; LoA: 0.59 ± 0.17 m·s^−1^
				Full	Mean velocity	*R*^2^: 0.72; LoA: 0.08 ± 0.19 m·s^−1^
					Peak velocity	*R*^2^: 0.65; LoA: 0.59 ± 0.32 m·s^−1^
		12 Camera raptor 3D motion capture	F/W Back Squat	40%1RM	Mean velocity	*R*^2^: 0.69; LoA: − 0.08 ± 0.09 m·s^−1^
					Peak velocity	*R*^2^: 0.91; LoA: 0.02 ± 0.14 m·s^−1^
				50%1RM	Mean velocity	*R*^2^: 0.95; LoA: − 0.06 ± 0.05 m·s^−1^
					Peak velocity	*R*^2^: 0.89; LoA: 0.07 ± 0.14 m·s^−1^
				60%1RM	Mean velocity	*R*^2^: 0.83; LoA: − 0.07 ± 0.08 m·s^−1^
					Peak velocity	*R*^2^: 0.84; LoA: 0.06 ± 0.17 m·s^−1^
				70%1RM	Mean velocity	*R*^2^: 0.84; LoA: − 0.07 ± 0.07 m·s^−1^
					Peak velocity	*R*^2^: 0.80; LoA: 0.09 ± 0.18 m·s^−1^
				80%1RM	Mean velocity	*R*^2^: 0.87; LoA: − 0.07 ± 0.09 m·s^−1^
					Peak velocity	*R*^2^: 0.60; LoA: 0.08 ± 0.28 m·s^−1^
				90%1RM	Mean velocity	*R*^2^: 0.92; LoA: − 0.07 ± 0.05 m·s^−1^
					Peak velocity	*R*^2^: 0.56; LoA: 0.08 ± 0.29 m·s^−1^
				100%1RM	Mean velocity	*R*^2^: 0.39; LoA: 0.10 ± 0.13 m·s^−1^
					Peak velocity	*R*^2^: 0.41; LoA: 0.01 ± 0.36 m·s^−1^
				Full	Mean velocity	*R*^2^: 0.97; LoA: − 0.07 ± 0.08 m·s^−1^
					Peak velocity	*R*^2^: 0.86; LoA: 0.06 ± 0.23 m·s^−1^
			Power clean	40%1RM	Mean velocity	*R*^2^: 0.62; LoA: 0.22 ± 0.18 m·s^−1^
					Peak velocity	*R*^2^: 0.59; LoA: 0.35 ± 0.43 m·s^−1^
				50%1RM	Mean velocity	*R*^2^: 0.54; LoA: 0.22 ± 0.22 m·s^−1^
					Peak velocity	*R*^2^: 0.11; LoA: 0.36 ± 0.62 m·s^−1^
				60%1RM	Mean velocity	*R*^2^: 0.50; LoA: 0.22 ± 0.15 m·s^−1^
					Peak velocity	*R*^2^: 0.68; LoA: 0.35 ± 0.26 m·s^−1^
				70%1RM	Mean velocity	*R*^2^: 0.24; LoA: 0.16 ± 0.26 m·s^−1^
					Peak velocity	*R*^2^: 0.08; LoA: 0.31 ± 0.74 m·s^−1^
				80%1RM	Mean velocity	*R*^2^: 0.35; LoA: 0.14 ± 0.22 m·s^−1^
					Peak velocity	*R*^2^: 0.06; LoA: 0.30 ± 0.64 m·s^−1^
				90%1RM	Mean velocity	*R*^2^: 0.41; LoA: 0.19 ± 0.15 m·s^−1^
					Peak velocity	*R*^2^: 0.26; LoA: 0.46 ± 0.35 m·s^−1^
				100%1RM	Mean velocity	*R*^2^: 0.23; LoA: 0.14 ± 0.16 m·s^−1^
					Peak velocity	*R*^2^: 0.60; LoA: 0.35 ± 0.23 m·s^−1^
				Full	Mean velocity	*R*^2^: 0.62; LoA: 0.18 ± 0.20 m·s^−1^
					Peak velocity	*R*^2^: 0.48; LoA: 0.35 ± 0.49 m·s^−1^
		12 Camera raptor 3D motion capture	F/W Back Squat	40%1RM	Mean velocity	*R*^2^: 0.82; LoA: 0.07 ± 0.12 m·s^−1^
					Peak velocity	*R*^2^: 0.96; LoA: 0.03 ± 0.12 m·s^−1^
				50%1RM	Mean velocity	*R*^2^: 0.75; LoA: 0.05 ± 0.16 m·s^−1^
					Peak velocity	*R*^2^: 0.93; LoA: 0.07 ± 0.16 m·s^−1^
			Power clean	60%1RM	Mean velocity	*R*^2^: 0.67; LoA: 0.04 ± 0.12 m·s^−1^
					Peak velocity	*R*^2^: 0.82; LoA: 0.12 ± 0.17 m·s^−1^
				70%1RM	Mean velocity	*R*^2^: 0.86; LoA: 0.04 ± 0.08 m·s^−1^
					Peak velocity	*R*^2^: 0.66; LoA: 0.19 ± 0.25 m·s^−1^
				80%1RM	Mean velocity	*R*^2^: 0.66; LoA: 0.05 ± 0.11 m·s^−1^
					Peak velocity	*R*^2^: 0.52; LoA: 0.31 ± 0.33 m·s^−1^
				90%1RM	Mean velocity	*R*^2^: 0.23; LoA: 0.01 ± 0.21 m·s^−1^
					Peak velocity	*R*^2^: 0.10; LoA: 0.31 ± 0.33 m·s^−1^
				100%1RM	Mean velocity	*R*^2^: 0.01; LoA: − 0.05 ± 0.18 m·s^−1^
					Peak velocity	*R*^2^: 0.02; LoA: 0.24 ± 0.49 m·s^−1^
				Full	Mean velocity	*R*^2^: 0.87; LoA: 0.03 ± 0.16 m·s^−1^
					Peak velocity	*R*^2^: 0.80; LoA: 0.18 ± 0.37 m·s^−1^
				40%1RM	Mean velocity	*R*^2^: 0.82; LoA: − 0.13 ± 0.14 m·s^−1^
					Peak velocity	*R*^2^: 0.47; LoA: − 0.36 ± 0.38 m·s^−1^
				50%1RM	Mean velocity	*R*^2^: 0.82; LoA: − 0.08 ± 0.15 m·s^−1^
					Peak velocity	*R*^2^: 0.22; LoA: − 0.25 ± 0.49 m·s^−1^
				60%1RM	Mean velocity	*R*^2^: 0.73; LoA: − 0.10 ± 0.18 m·s^−1^
					Peak velocity	*R*^2^: 0.61; LoA: − 0.20 ± 0.35 m·s^−1^
				70%1RM	Mean velocity	*R*^2^: 0.04; LoA: − 0.02 ± 0.34 m·s^−1^
					Peak velocity	*R*^2^: 0.50; LoA: − 0.15 ± 0.38 m·s^−1^
				80%1RM	Mean velocity	*R*^2^: 0.07; LoA: 0.01 ± 0.27 m·s^−1^
					peak velocity	*R*^2^: 0.69; LoA: − 0.12 ± 0.27 m·s^−1^
				90%1RM	Mean velocity	*R*^2^: 0.18; LoA: 0.02 ± 0.22 m·s^−1^
					Peak velocity	*R*^2^: 0.84; LoA: − 0.04 ± 0.18 m·s^−1^
				100%1RM	Mean velocity	*R*^2^: 0.02; LoA: 0.01 ± 0.27 m·s^−1^
					Peak velocity	*R*^2^: 0.57; LoA: − 0.05 ± 0.27 m·s^−1^
					Mean velocity	*R*^2^: 0.73; LoA: − 0.04 ± 0.25 m·s^−1^
				Full	Peak velocity	*R*^2^: 0.74; LoA: − 0.17 ± 0.39 m·s^−1^
		12 Camera raptor 3D motion capture	F/W Back Squat	40%1RM	Mean velocity	*R*^2^: 0.64; LoA: − 0.01 ± 0.16 m·s^−1^
					Peak velocity	*R*^2^: 0.10; LoA: − 0.05 ± 0.50 m·s^−1^
				50%1RM	Mean velocity	*R*^2^: 0.71; LoA: 0.04 ± 0.13 m·s^−1^
					Peak velocity	*R*^2^: 0.12; LoA: − 0.04 ± 0.43 m·s^−1^
				60%1RM	Mean velocity	*R*^2^: 0.49; LoA: 0.08 ± 0.21 m·s^−1^
					Peak velocity	*R*^2^: 0.00; LoA: 0.02 ± 0.53 m·s^−1^
				70%1RM	Mean velocity	*R*^2^: 0.46; LoA: 0.11 ± 0.18 m·s^−1^
					Peak velocity	*R*^2^: 0.00; LoA: 0.01 ± 0.56 m·s^−1^
				80%1RM	Mean velocity	*R*^2^: 0.58; LoA: 0.16 ± 0.18 m·s^−1^
					Peak velocity	*R*^2^: 0.02; LoA: 0.04 ± 0.44 m·s^−1^
				90%1RM	Mean velocity	*R*^2^: 0.12; LoA: 0.10 ± 0.22 m·s^−1^
					Peak velocity	*R*^2^: 0.58; LoA: 0.22 ± 0.28 m·s^−1^
				100%1RM	Mean velocity	*R*^2^: 0.20; LoA: − 0.09 ± 0.26 m·s^−1^
					Peak velocity	*R*^2^: 0.15; LoA: 0.25 ± 0.52 m·s^−1^
				Full	Mean velocity	*R*^2^: 0.80; LoA: 0.06 ± 0.24 m·s^−1^
					Peak velocity	*R*^2^: 0.57; LoA: 0.06 ± 0.51 m·s^−1^
van den Tillaar and Ball [11]	PUSH Band	ET-Enc-02 Ergotest linear encoder	F/W Bench Press	50%1RM, + 10 to 30 kg	Mean velocity	Bias: 0.11 m·s^−1^; SEE: 0.17 m·s^−1^; *r*:0.62
					Mean peak velocity	Bias: 0.22 m·s^−1^; SEE: 0.33 m·s^−1^; *r*:0.49
			Push-Up	Body Weight, 10–30 kg Weight vests	Mean velocity	Bias: 0.12 m·s^−1^; SEE: 0.16 m·s^−1^; *r*: 0.70
					Mean peak velocity	Bias: 0.15 m·s^−1^; SEE: 0.34 m·s^−1^; *r*: 0.46

*1RM* one repetition maximum, *SEE* standard error of the estimate, *MV* mean concentric velocity, *T-Force* T-force linear velocity transducer, *GymAware* GymAware PowerTool, *CV* coefficient of variation, *RMSE* root mean square of the estimate, *LPR* least products regression, *TEE* typical error of the estimate, *S/M* Smith machine, *F/W* free weight, *MD* mean difference, *r* Pearson’s correlation coefficient, *ICC* intraclass correlation coefficient, SEM standard error of measurement, CMJ countermovement jump, *ES* effect size, *ROM* range of motion, *RTE* relative typical error, *OH* overhead, *DB* dumbbell

**Table 6:** Summary of studies that investigated the validity of mobile phone and tablet applications used to measure kinetic and kinematic variables during resistance training

Study	Device	Criterion	Exercise	Intensity/load	Variable measured	Findings
Balsalobre-Fernández et al. [69]	PowerLift (v4.0 iOS)	SmartCoach Power Encoder	F/W Back Squat	50–95%1RM	Mean velocity	Bias: − 0.005 ± 0.04 m·s^−1^
						SEE: 0.04 m·s^−1^
			F/W Bench Press	50–95%1RM	Mean velocity	Bias: − 0.01 ± 0.05 m·s^−1^
						SEE: 0.05 m·s^−1^
			F/W Hip-Thrust	50–95%1RM	Mean velocity	Bias: 0.02 ± 0.04 m·s^−1^
						SEE: 0.03 m·s^−1^
Balsalobre-Fernández et al. [70]	PowerLift (v2.8 iOS)	SmartCoach Power Encoder	F/W Bench Press	75–100%1RM	Mean velocity	*r: *0.94; SEE: 0.028 m·s^−1^; ICC: 0.965
						MD: 0.008± 0.03 m·s^−1^
Courel-Ibanez et al. [36]	PowerLift (v4.0 iOS)	T-Force	S/M Bench Press	20–80 kg	Mean velocity	SEM: 0.09 m·s^−1^; CV: 11.7%; ICC: 0.966
			S/M Back Squat	20–80 kg	Mean velocity	SEM: 0.06 m·s^−1^; CV: 7.6%; ICC: 0.955
de Sá et al. [80]	iLoad App (v 1.0)	Chronojump	S/M Half ROM Back Squat	10RM	Mean velocity	Bias ± random error: − 0.022 ± 0.034 m·s^−1^
						ES: − 0.21 (− 0.39, − 0.04); *r*: 0.948
				10RM	Total work	Bias ± random error: 0.706 ± 3.391 kJ
						ES: 0.04 (− 0.07, 0.16); *r*: 0.977
Martinez-Cava et al. [51]	My Lift (v8.1 iOS)	T-Force	S/M Back Squat	25–95 kg	Peak velocity	SEM: 0.12 m·s^−1^; CV: 7.59%; ICC: 0.937
			S/M Bench Press	25–95 kg	Peak velocity	SEM: 0.10 m·s^−1^; CV: 7.04%; ICC: 0.991
Perez-Castilla et al. [10]	PowerLift (v.6.0.1 iOS)	OptiTrack	S/M Bench Press	55–85%1RM	Mean velocity	Bias: − 0.01 ± 0.05 m·s^−1^
Sanchez-Pay et al. [23]	Kinovea (v0.8.15) via	T-Force	S/M Bench Press	All loads	Mean velocity	Bias: 0.10 ± 0.06 m·s^−1^; *r: *0.997
	Samsung S6				Distance	Bias: 1.07 ± 0.65 cm; *r: *0.996
					Time	Bias: − 61.6 ± 36.1 ms; *r: *0.998
				High Loads (MV < 0.80 m·s^−1^)	Mean velocity	Bias: 0.06 ± 0.05 m·s^−1^; *r: *0.986
					Distance	Bias: − 0.95 ± 0.69 cm; *r: *0.992
					Time	Bias: − 76.6 ± 41.3 ms; *r: *0.997
				Low Loads (MV > 0.80 m·s^−1^)	Mean velocity	Bias: 0.14 ± 0.06 m·s^−1^; *r: *0.985
					Distance	Bias: 1.21 ± 0.59 cm; *r: *0.996
					Time	Bias: − 44.2 ± 17.2 ms; *r: *0.978
	Kinovea (v0.8.15) via	T-Force	S/M Bench Press	All Loads	Mean velocity	Bias: 0.09 ± 0.06 m·s^−1^; *r: *0.996
	Xiaomi A1				Distance	Bias: 1.24 ± 0.45 cm; *r: *0.998
					Time	Bias: − 57.4 ± 33.4 ms; *r: *0.998
				High Loads (MV < 0.80 m·s^−1^)	Mean velocity	Bias: 0.06 ± 0.03 m·s^−1^; *r: *0.994
					Distance	Bias: 1.21 ± 0.47 cm; *r: *0.995
					Time	Bias: − 74.3 ± 34.5 ms; *r: *0.998
				Low Loads (MV > 0.80 m·s^−1^)	Mean velocity	Bias: 0.13 ± 0.06 m·s^−1^; *r: *0.981
					Distance	Bias: 1.38 ± 0.39 cm; *r: *0.997
					Time	Bias: − 37.6 ± 18.0 ms; *r: *0.975
	Kinovea (v0.8.15) via iPhone X	T-Force	S/M Bench Press	All Loads	Mean velocity	Bias: 0.11 ± 0.08 m·s^−1^; *r*: 0.994
					Distance	Bias: 1.34 ± 0.75 cm; *r*: 0.993
					Time	Bias: − 69.5 ± 34.8 ms; * r*: 0.998
				High Loads (MV < 0.80 m·s^−1^)	Mean velocity	Bias: 0.07 ± 0.04 m·s^−1^;* r*: 0.995
					Distance	Bias: 1.29 ± 0.81 cm; * r*: 0.986
					Time	Bias: − 85.6 ± 41.3 ms;* r*: 0.998
				Low Loads (MV > 0.80 m·s^−1^)	Mean velocity	Bias: 0.17 ± 0.09 m·s^−1^;* r*: 0.977
					Distance	Bias: 1.40 ± 0.68 cm; *r*: 0.993
					Time	Bias: − 50.4 ± 22.0 ms; *r:* 0.961
	Kinovea (v0.8.15) via Casio FH20	T-Force	S/M Bench Press	All Loads	Mean velocity	Bias: 0.14 ± 0.09 m·s^−1^; *r:* 0.992
					Distance	Bias: 2.48 ± 0.87 cm; *r:* 0.990
					Time	Bias: − 69.5 ± 40.7 ms; *r:* 0.997
				High Loads (MV < 0.80 m·s^−1^)	Mean velocity	Bias: 0.08 ± 0.05 m·s^−1^; *r:* 0.990
					Distance	Bias: 2.36 ± 0.91 cm; *r:* 0.984
					Time	Bias: − 83.4 ± 44.3 ms; *r:* 0.996
				Low Loads (MV > 0.80 m·s^−1^)	Mean velocity	Bias: 0.20 ± 0.09 m·s^−1^; *r:* 0.963
					Distance	Bias: 2.62 ± 0.81 cm; *r:* 0.989
					Time	Bias: − 53.2 ± 28.9 ms; *r:* 0.932
Sanudo et al. [81]	Kinovea (v0.8.15) via Digital Video Camera (50Hz)	T-Force	S/M Bench Press	20 kg	Mean propulsive velocity	Bias: − 0.43 m·s^−1^
					Maximal velocity	Bias: − 0.57 m·s^−1^
				30 kg	Mean propulsive velocity	Bias: − 0.41 m·s^−1^
					Maximal velocity	Bias: − 0.59 m·s^−1^
				40 kg	Mean propulsive velocity	Bias: − 0.30 m·s^−1^
					Maximal velocity	Bias: − 0.42 m·s^−1^
				50 kg	Mean propulsive velocity	Bias: − 0.23 m·s^−1^
					Maximal velocity	Bias: − 0.36 m·s^−1^
				60 kg	Mean propulsive velocity	Bias: − 0.16 m·s^−1^
					Maximal velocity	Bias: − 0.28 m·s^−1^
				70 kg	Mean propulsive velocity	Bias: − 0.14 m·s^−1^
					Maximal velocity	Bias: − 0.28 m·s^−1^
				80 kg	Mean propulsive velocity	Bias: − 0.16 m·s^−1^
					Maximal velocity	Bias: − 0.23 m·s^−1^
Thompson et al. [24]	MyLift (PowerLift at the time of data collection)	12 Camera raptor 3D motion capture	F/W Back Squat	40%1RM	Mean velocity	*R*^2^: 0.96; LoA: 0.02 ± 0.06 m·s^−1^
				50%1RM	Mean velocity	*R*^2^: 0.94; LoA: 0.01 ± 0.05 m·s^−1^
				60%1RM	Mean velocity	*R*^2^: 0.88; LoA: 0.01 ± 0.07 m·s^−1^
				70%1RM	Mean velocity	*R*^2^: 0.95; LoA: 0.01 ± 0.04 m·s^−1^
				80%1RM	Mean velocity	*R*^2^: 0.93; LoA: 0.00 ± 0.05 m·s^−1^
				90%1RM	Mean velocity	*R*^2^: 0.92; LoA: 0.00 ± 0.04 m·s^−1^
				100%1RM	Mean velocity	*R*^2^: 0.85; LoA: 0.00 ± 0.06 m·s^−1^
				Full	Mean velocity	*R*^2^: 0.99; LoA: 0.01 ± 0.05 m·s^−1^

*1RM* one repetition maximum, *MV* mean concentric velocity, *CV* coefficient of variation, *T-Force* T-force linear velocity transducer, *S/M* Smith machine, *F/W* free weight, *r* Pearson’s correlation coefficient, *ES* effect size, *ROM* range of motion, *RE* random error, *SEE* standard error of the estimate, *SEM* standard error of the measurement

**Table 7:** Summary of studies that investigated the validity of optic devices used to measure kinetic and kinematic variables during resistance training

Study	Device	Criterion	Exercise	Intensity/load	Variable measured	Findings
Courel-Ibanez et al. [36]	Velowin	T-Force	S/M Bench Press	20–80 kg	Mean velocity	SEM: 0.02 m·s^−1^; CV: 3.1%; ICC: 0.998
					Mean propulsive velocity	SEM: 0.03 m·s^−1^; CV: 3.5%; ICC: 0.997
					Peak velocity	SEM: 0.02 m·s^−1^; CV: 1.7%; ICC: 0.999
			S/M Back Squat	20–80 kg	Mean velocity	SEM: 0.03 m·s^−1^; CV: 4.4%; ICC: 0.992
					Mean propulsive velocity	SEM: 0.03 m·s^−1^; CV: 3.6%; ICC: 0.992
					Peak velocity	SEM: 0.04 m·s^−1^; CV: 2.3%; ICC: 0.993
			S/M Prone Bench Pull	20–80 kg	Mean velocity	SEM: 0.09 m·s^−1^; CV: 8.1%; ICC: 0.967
					Mean propulsive velocity	SEM: 0.25 m·s^−1^; CV: 8.1%; ICC: 0.967
					Peak velocity	SEM: 0.03 m·s^−1^; CV: 1.9%; ICC: 0.999
Garcia-Ramos et al. [71]	Velowin	T-Force	F/W Back Squat	20–70 kg	Mean velocity	Bias: 0.02 ± 0.05 m·s^−1^; SEE: 0.040 m·s^−1^
					Mean propulsive velocity	Bias: 0.02 ± 0.06 m·s^−1^; SEE: 0.055 m·s^−1^
					Maximum velocity	Bias: − 0.09 ± 0.06 m·s^−1^; SEE: 0.057 m·s^−1^
Laza-Cagigas et al. [68]	Velowin	Oqus Infrared cameras and kistler multicomponent force platform	F/W Back Squat	< 30–90%1RM	Displacement	RMSE: 3.73 cm; CV: 6.6%; ICC: 0.84
					Mean velocity	RMSE: 0.06 m·s^−1^; CV: 7.3%; ICC: 0.97
					Peak velocity	RMSE: 0.09 m·s^−1^; CV: 6.5%; ICC: 0.96
					Mean force	RMSE: 43N; CV: 3.6%; ICC: 0.99
					Peak force	RMSE: 100N; CV: 5.2%; ICC: 0.98
					Mean power	RMSE: 73W; CV: 8.2%; ICC: 0.92
					Peak power	RMSE: 160W; CV: 8.3%; ICC: 0.85
Peña Garcia-Orea et al. [82]	Velowin	T-Force	S/M Squat	20–70 kg	Mean velocity	No significant differences were found between the variances of the two devices
					Mean propulsive velocity	
					Peak velocity	
Peña Garcia-Orea et al. [72]	Velowin	T-Force	Loaded CMJ	3.5–43.5 kg	Mean velocity	No significant differences were found between the variances of the two devices
					Peak velocity	
Perez-Castilla et al. [10]	Velowin	OptiTrack	S/M Bench Press	55–85%1RM	Mean velocity	Bias: − 0.05 ± 0.03 m·s^−1^
Weakley et al. [22]	FLEX	Vicon	F/W Back Squat	20%1RM	Mean velocity	Bias: 0.00 ± 0.01 m·s^−1^; TEE: 0.06 ± 0.02 m·s^−1^; *r:* 0.97 ± 0.03
				40%1RM	Mean velocity	Bias: 0.00 ± 0.00 m·s^−1^; TEE: 0.02 ± 0.008 m·s^−1^; *r:* 0.99 ± 0.01
				60%1RM	Mean velocity	Bias: 0.00 ± 0.00 m·s^−1^; TEE: 0.02 ± 0.008 m·s^−1^; *r: *0.97 ± 0.03
				80%1RM	Mean velocity	Bias: 0.00 ± 0.00 m·s^−1^; TEE: 0.02 ± 0.004 m·s^−1^; *r:* 0.95 ± 0.05
				≥ 90%1RM	Mean velocity	Bias: 0.00 ± 0.00 m·s^−1^; TEE: 0.02 ± 0.004 m·s^−1^; *r:* 0.99 ± 0.01
				Overall	Mean velocity	Bias: 0.00 ± 0.00 m·s^−1^; TEE: 0.03 ± 0.004 m·s^−1^; *r:* 0.99 ± 0.00
			F/W Bench Press	20%1RM	Mean velocity	Bias: − 0.01 ± 0.03 m·s^−1^; TEE: 0.08 ± 0.04 m·s^−1^; *r:* 0.97 ± 0.04
				40%1RM	Mean velocity	Bias: − 0.02 ± 0.06 m·s^−1^; TEE: 0.04 ± 0.02 m·s^−1^; *r:* 0.99 ± 0.02
				60%1RM	Mean velocity	Bias: 0.00 ± 0.01 m·s^−1^; TEE: 0.02 ± 0.04 m·s^−1^; *r:* 0.98 ± 0.02
				80%1RM	Mean velocity	Bias: 0.00 ± 0.00 m·s^−1^; TEE: 0.01 ± 0.005 m·s^−1^; *r:* 0.99 ± 0.01
				≥ 90%1RM	Mean velocity	Bias: 0.00 ± 0.00 m·s^−1^; TEE: 0.02 ± 0.005 m·s^−1^; *r:* 0.98 ± 0.01
				Overall	Mean velocity	Bias: − 0.01 ± 0.01 m·s^−1^; TEE: 0.04 ± 0.005 m·s^−1^; *r:* 0.99 ± 0.00

*1RM* one repetition maximum, *SEE* standard error of the estimate, *T-force* T-force linear velocity transducer, *CV* coefficient of variation, *RMSE* root mean square of the estimate, *TEE* typical error of the estimate, *S/M* Smith machine, *F/W* free weight, *r* Pearson’s correlation coefficient, *ICC* intraclass correlation coefficient, *SEM* standard error of measurement, *CMJ* countermovement jump

**Table 8:** Summary of studies that investigated the reliability of linear transducer devices used to measure kinetic and kinematic variables during resistance training

Study	Device	Reliability	Exercise	Intensity/load	Variable Measured	Findings
Balsalobre-Fernández et al. [62]	T-Force	Intra-device	S/M Back Squat	20–70 kg	Peak velocity	CV: 4.2 ± 2.5%; ICC: 0.988; *r: *0.975
					Mean velocity	CV: 3.9 ± 2.4%; ICC: 0.989; *r:* 0.989
Balsalobre-Fernández et al. [69]	SmartCoach	Intra-device	F/W Back Squat	50–95%1RM	Mean velocity	ICC: 0.981
			F/W Bench Press	50–95%1RM	Mean velocity	ICC: 0.981
			F/W Hip Thrust	50–95%1RM	Mean velocity	ICC: 0.966
Beckham et al. [28]	GymAware	Intra-device	F/W Back Squat	45%1RM	Mean velocity	ICC: 0.774
					Peak velocity	ICC: 0.793
				60%1RM	Mean velocity	ICC: 0.752
					Peak velocity	ICC: 0.775
				75%1RM	Mean velocity	ICC: 0.651
					Peak velocity	ICC: 0.761
Boehringer and Whyte [77]	1080Q	Intra-device	S/M Bench Press	30–80%1RM	Mean velocity	MD: 0.004 m·s^−1^; CV:7.0%; ICC: 0.97
				30%1RM		MD: − 0.003 m·s^−1^; CV: 4.5%; ICC: 0.64
				40%1RM		MD: 0.001 m·s^−1^; CV: 3.7; ICC: 0.82
				50%1RM		MD: 0.010 m·s^−1^; CV: 5.7 ICC: 0.53
				60%1RM		MD: 0.017 m·s^−1^; CV: 6.5 ICC: 0.45
				70%1RM		MD: 0.000 m·s^−1^; CV: 7.6 ICC: 0.63
				80%1RM		MD: − 0.003 m·s^−1^; CV: 11.0 ICC: 0.69
				30–80%1RM	Mean force	MD: − 0.3N; CV: 1.7%; ICC: 1.00
				30%1RM		MD: − 4.1N; CV: 2.0%; ICC: 0.93
				40%1RM		MD: − 0.9N; CV: 1.9%; ICC: 0.98
				50%1RM		MD: 3.4N; CV: 2.3%; ICC: 0.96
				60%1RM		MD: 3.2N; CV: 1.7%; ICC: 0.98
				70%1RM		MD: − 4.8N; CV: 1.1%; ICC: 0.99
				80%1RM		MD: − 0.9N; CV: 0.9%; ICC: 0.99
				30–80%1RM	Mean power	MD: 3.4W; CV: 8.0%; ICC: 0.90
				30%1RM		MD: − 4.1W; CV: 6.7%; ICC: 0.73
				40%1RM		MD: − 1.6W; CV: 5.1%; ICC: 0.91
				50%1RM		MD: 8.1W; CV: 7.3%; ICC: 0.79
				60%1RM		MD: 14.8W; CV: 7.6%; ICC: 0.74
				70%1RM		MD: − 0.4W; CV: 8.3%; ICC: 0.73
				80%1RM		MD: − 1.1W; CV: 11.4%; ICC: 0.65
				30–80%1RM	Peak velocity	MD: 0.002 m·s^−1^; CV: 6.3%; ICC: 0.97
				30%1RM		MD: 0.002 m·s^−1^; CV: 4.1%; ICC: 0.61
				40%1RM		MD: 0.001 m·s^−1^; CV: 3.6%; ICC: 0.84
				50%1RM		MD: 0.008 m·s^−1^; CV: 5.0%; ICC: 0.68
				60%1RM		MD: 0.000 m·s^−1^; CV: 5.0%; ICC: 0.75
				70%1RM		MD: 0.004 m·s^−1^; CV: 8.2%; ICC: 0.58
				80%1RM		MD: − 0.001 m·s^−1^; CV: 9.5%; ICC: 0.80
				30–80%1RM	Peak force	MD: − 2.6N; CV: 4.4%; ICC: 0.94
				30%1RM		MD: − 20.9N; CV: 7.0%; ICC: 0.51
				40%1RM		MD: − 4.9N; CV: 3.5%; ICC: 0.90
				50%1RM		MD: 7.4N; CV: 4.9%; ICC: 0.79
				60%1RM		MD: 1.3N; CV: 4.2%; ICC: 0.80
				70%1RM		MD: 0.4N; CV: 4.3%; ICC: 0.78
				80%1RM		MD: − 9.4N; CV: 3.7%; ICC: 0.87
				30–80%1RM	Peak power	MD: 2.0W; CV: 7.4%; ICC: 0.91
				30%1RM		MD: 2.4W; CV: 6.1%; ICC: 0.79
				40%1RM		MD: − 2.2W; CV: 5.1%; ICC: 0.92
				50%1RM		MD: 6.9W; CV: 7.0%; ICC: 0.85
				60%1RM		MD: 1.3W; CV: 6.1%; ICC: 0.88
				70%1RM		MD: 1.6W; CV: 8.9%; ICC: 0.77
				80%1RM		MD: 2.5W; CV: 10.2%; ICC: 0.79
Courel-Ibanez et al. [36]	T-Force	Inter-device	S/M Bench Press	20–80 kg	Mean velocity	SEM: 0.01 m·s^−1^; CV: 1.4%; ICC: 1.000
					Mean propulsive velocity	SEM: 0.01 m·s^−1^; CV: 1.3%; ICC: 1.000
					Peak velocity	SEM: 0.01 m·s^−1^; CV: 0.6%; ICC: 1.000
			S/M Back Squat	20–80 kg	Mean velocity	SEM: 0.01 m·s^−1^; CV: 1.0%; ICC: 0.999
					Mean propulsive velocity	SEM: 0.01 m·s^−1^; CV: 1.1%; ICC: 0.999
					Peak velocity	SEM: 0.01 m·s^−1^; CV: 0.8%; ICC: 0.999
			S/M Prone Bench Pull	20–80 kg	Mean velocity	SEM: 0.02 m·s^−1^; CV: 2.1%; ICC: 0.998
					Mean propulsive velocity	SEM: 0.02 m·s^−1^; CV: 1.9%; ICC: 0.998
					Peak velocity	SEM: 0.01 m·s^−1^; CV: 0.8%; ICC: 1.000
	Chronojump	Inter-device	S/M Bench Press	20–80 kg	Mean velocity	SEM: 0.04 m·s^−1^; CV: 4.7%; ICC: 0.995
					Mean propulsive velocity	SEM: 0.04 m·s^−1^; CV: 5.2%; ICC: 0.995
					Peak velocity	SEM: 0.02 m·s^−1^; CV: 1.4%; ICC: 1.000
				20–80 kg	Mean velocity	SEM: 0.03 m·s^−1^; CV: 3.6%; ICC: 0.991
					Mean propulsive velocity	SEM: 0.03 m·s^−1^; CV: 3.9%; ICC: 0.991
			S/M Back Squat		Peak velocity	SEM: 0.03 m·s^−1^; CV: 1.8%; ICC: 0.996
				20–80 kg	Mean velocity	SEM: 0.04 m·s^−1^; CV: 3.3%; ICC: 0.995
					Mean propulsive velocity	SEM: 0.04 m·s^−1^; CV: 3.4%; ICC: 0.995
			S/M Prone Bench Pull		Peak velocity	SEM: 0.04 m·s^−1^; CV: 2.4%; ICC: 1.00
	T-Force	Intra-device	S/M Bench Press	20–80 kg	Mean velocity	SEM: 0.02 m·s^−1^; CV: 1.9%; ICC: 0.999
					Mean propulsive velocity	SEM: 0.02 m·s^−1^; CV: 1.8%; ICC: 0.999
					Peak velocity	SEM: 0.03 m·s^−1^; CV: 2.0%; ICC: 0.999
			S/M Back Squat	20–80 kg	Mean velocity	SEM: 0.03 m·s^−1^; CV: 2.5%; ICC: 0.995
					Mean propulsive velocity	SEM: 0.02 m·s^−1^; CV: 2.6%; ICC: 0.996
					Peak velocity	SEM: 0.05 m·s^−1^; CV: 2.9%; ICC: 0.989
			S/M Prone Bench Pull	20–80 kg	Mean velocity	SEM: 0.04 m·s^−1^; CV: 3.0%; ICC: 0.995
					Mean propulsive velocity	SEM: 0.03 m·s^−1^; CV: 3.0%; ICC: 0.995
					Peak velocity	SEM: 0.03 m·s^−1^; CV: 1.8%; ICC: 0.999
	Chronojump	Intra-device	S/M Bench Press	20–80 kg	Mean velocity	SEM: 0.04 m·s^−1^; CV: 4.3%; ICC: 0.997
					Mean propulsive velocity	SEM: 0.03 m·s^−1^; CV: 3.2%; ICC: 0.998
					Peak velocity	SEM: 0.04 m·s^−1^; CV: 2.4%; ICC: 0.999
			S/M Back Squat	20–80 kg	Mean velocity	SEM: 0.04 m·s^−1^; CV: 3.9%; ICC: 0.990
					Mean propulsive velocity	SEM: 0.04 m·s^−1^; CV: 3.8%; ICC: 0.991
					Peak velocity	SEM: 0.06 m·s^−1^; CV: 3.4%; ICC: 0.985
			S/M Prone Bench Pull	20–80 kg	Mean velocity	SEM: 0.07 m·s^−1^; CV: 5.2%; ICC: 0.990
					Mean propulsive velocity	SEM: 0.07 m·s^−1^; CV: 5.4%; ICC: 0.987
					Peak velocity	SEM: 0.04 m·s^−1^; CV: 2.3%; ICC: 0.998
Dorrell et al. [26]	GymAware	Intra-device	F/W Back Squat	80%1RM	Bar displacement	Mean TE: 3.8% (3.0–5.3%)
					Peak velocity	Mean TE: 8.1% (6.4–11.5%)
					Mean velocity	Mean TE: 7.0% (5.6–10.0%)
					Peak force	Mean TE: 4.3% (3.4–6.1%)
					Mean force	Mean TE: 0.6% (0.5–0.9%)
			F/W Bench Press	80%1RM	Bar displacement	Mean TE: 3.0% (2.3–4.1)
					Peak velocity	Mean TE: 6.2% (4.9–8.7)
					Mean velocity	Mean TE: 7.4% (5.8–10.5%)
			F/W Deadlift	80%1RM	Bar displacement	Mean TE: 2.0% (1.6–2.7%)
					Peak velocity	Mean TE: 8.8% (7.0–12.5%)
					Mean velocity	Mean TE: 7.0% (5.5–9.8%)
					Peak force	Mean TE: 3.1% (2.5–4.4%)
					Mean force	Mean TE: 1.6% (1.3–2.2%)
Fernandes et al. [55]	FitroDyne (fitronic)	Intra-device (intra-day)	S/M Bench Press	20%1RM	Peak power	TE: 18.3W; CV: 4.3%
					Mean power	TE: 8.2W; CV: 3.4%
					Peak velocity	TE: 6.9 cm·s^−1^; CV: 3.3%
					Mean velocity	TE: 3.4 cm·s^−1^; CV: 2.9%
				30%1RM	Peak power	TE: 11.3W; CV: 2.1%
					Mean power	TE: 8.4W; CV: 2.6%
					Peak velocity	TE: 3.1 cm·s^−1^; CV: 1.7%
					Mean velocity	TE: 2.9 cm·s^−1^; CV: 2.7%
				40%1RM	Peak power	TE: 10.1W; CV: 1.6%
					Mean power	TE: 11.0W; CV: 2.9%
					Peak velocity	TE: 2.8 cm·s^−1^; CV: 1.8%
					Mean velocity	TE: 2.9 cm·s^−1^; CV: 3.1%
				50%1RM	Peak power	TE: 16.9W; CV: 2.6%
					Mean power	TE: 10.7W; CV: 2.7%
					Peak velocity	TE: 3.6 cm·s^−1^; CV: 2.8%
					Mean velocity	TE: 2.2 cm·s^−1^; CV: 2.8%
				60%1RM	Peak power	TE: 23.8W; CV: 4.0%
					Mean power	TE: 17.7W; CV: 4.6%
					Peak velocity	TE: 3.8 cm·s^−1^; CV: 3.9%
					Mean velocity	TE: 2.8 cm·s^−1^; CV: 4.4%
				70%1RM	Peak power	TE: 18.6W; CV: 3.4%
					Mean power	TE: 16.4W; CV: 4.8%
					Peak velocity	TE: 2.6 cm·s^−1^; CV: 3.3%
					Mean velocity	TE: 2.2 cm·s^−1^; CV: 4.5%
				80%1RM	Peak power	TE: 53.0W; CV: 12.2%
					Mean power	TE: 44.2W; CV: 17.1%
					Peak velocity	TE: 5.3 cm·s^−1^; CV: 9.7%
					Mean velocity	TE: 4.3 cm·s^−1^; CV: 13.4%
			S/M Back Squat	20%1RM	Peak power	TE: 21.8W; CV:4.9 %
					Mean power	TE: 13.3W; CV: 5.3%
					Peak velocity	TE: 7.0 cm·s^−1^; CV: 4.1%
					Mean velocity	TE: 3.9 cm·s^−1^; CV: 4.0%
				30%1RM	Peak power	TE: 34.6W; CV: 5.4%
					Mean power	TE: 19.9W; CV: 5.6%
					Peak velocity	TE: 7.4 cm·s^−1^; CV: 4.5%
					Mean velocity	TE: 4.2 cm·s^−1^; CV: 4.6%
				40%1RM	Peak power	TE: 35.2W; CV: 4.3%
					Mean power	TE: 28.2W; CV: 6.4%
					Peak velocity	TE: 6.1 cm·s^−1^; CV: 4.0%
					Mean velocity	TE: 4.8 cm·s^−1^; CV: 5.7%
				50%1RM	Peak power	TE: 22.8W; CV: 2.4%
					Mean power	TE: 14.2W; CV: 2.9%
					Peak velocity	TE: 3.9 cm·s^−1^; CV: 2.7%
					Mean velocity	TE: 2.4 cm·s^−1^; CV: 3.2%
				60%1RM	Peak power	TE: 36.7W; CV: 3.5%
					Mean power	TE: 22.3W; CV: 4.1%
					Peak velocity	TE: 4.5 cm·s^−1^; CV: 3.3%
					Mean velocity	TE: 3.0 cm·s^−1^; CV: 4.3%
				70%1RM	Peak power	TE: 55.6W; CV: 4.9%
					Mean power	TE: 29.6W; CV: 5.4%
					Peak velocity	TE: 6.4 cm·s^−1^; CV: 5.2%
					Mean velocity	TE: 3.8 cm·s^−1^; CV: 6.3%
				80%1RM	Peak power	TE: 46.7W; CV: 3.9%
					Mean power	TE: 28.4W; CV: 5.5%
					Peak velocity	TE: 5.1 cm·s^−1^; CV: 4.4%
					Mean velocity	TE: 3.1 cm·s^−1^; CV: 6.4%
			S/M Bent Over Row	20%1RM	Peak power	TE: 18.9W; CV: 5.1%
					Mean power	TE: 14.5W; CV: 6.6%
					Peak velocity	TE: 8.8 cm·s^−1^; CV: 4.4%
					Mean velocity	TE: 6.6 cm·s^−1^; CV: 5.6%
				30%1RM	Peak power	TE: 20.0W; CV: 3.8%
					Mean power	TE: 23.0W; CV: 7.4%
					Peak velocity	TE: 7.1 cm·s^−1^; CV: 3.8%
					Mean velocity	TE: 8.7 cm·s^−1^; CV: 7.9%
				40%1RM	Peak power	TE: 21.6W; CV: 3.5%
					Mean power	TE: 16.3W; CV: 4.3%
					Peak velocity	TE: 6.1 cm·s^−1^; CV: 3.7%
					Mean velocity	TE: 4.8 cm·s^−1^; CV: 4.7%
				50%1RM	Peak power	TE: 20.8W; CV: 2.9%
					Mean power	TE: 26.7W; CV: 6.1%
					Peak velocity	TE: 4.0 cm·s^−1^; CV: 2.6%
					Mean velocity	TE: 6.0 cm·s^−1^; CV: 6.4%
				60%1RM	Peak power	TE: 33.0W; CV: 4.1%
					Mean power	TE: 33.0W; CV: 6.9%
					Peak velocity	TE: 5.7 cm·s^−1^; CV: 4.0%
					Mean velocity	TE: 6.4 cm·s^−1^; CV: 7.5%
				70%1RM	Peak power	TE: 62.8W; CV: 7.8%
					Mean power	TE: 40.0W; CV: 8.2%
					Peak velocity	TE: 10.5 cm·s^−1^; CV: 8.5%
					Mean velocity	TE: 6.7 cm·s^−1^; CV: 9.0%
				80%1RM	Peak power	TE: 61.1W; CV: 7.7%
					Mean power	TE: 37.3W; CV: 7.8%
					Peak velocity	TE: 8.8 cm·s^−1^; CV: 8.3%
					Mean velocity	TE: 5.4 cm·s^−1^; CV: 8.5%
Garcia-Pinillos et al. [61]	T-Force	Intra-device	S/M Concentric-Only Half Back Squat	10%1RM	Mean velocity	CV: 8.60%; SEM: 0.03 m·s^−1^
					Mean propulsive velocity	CV: 11.28%; SEM: 0.04 m·s^−1^
					Maximum velocity	CV: 9.18%; SEM: 0.06 m·s^−1^
				20%1RM	Mean velocity	CV: 6.76%; SEM: 0.02 m·s^−1^
					Mean propulsive velocity	CV: 8.29%; SEM: 0.03 m·s^−1^
					Maximum velocity	CV: 8.17%; SEM: 0.05 m·s^−1^
				30%1RM	Mean velocity	CV: 11.86%; SEM: 0.11 m·s^−1^
					Mean propulsive velocity	CV: 14.39%; SEM: 0.04 m·s^−1^
					Maximum velocity	CV: 7.42%; SEM: 0.04 m·s^−1^
				40%1RM	Mean velocity	CV: 9.95%; SEM: 0.09 m·s^−1^
					Mean propulsive velocity	CV: 10.87%; SEM: 0.03 m·s^−1^
					Maximum velocity	CV: 6.64%; SEM: 0.03 m·s^−1^
				50%1RM	Mean velocity	CV: 9.06%; SEM: 0.07 m·s^−1^
					Mean propulsive velocity	CV: 11.21%; SEM: 0.02 m·s^−1^
					Maximum velocity	CV: 6.49%; SEM: 0.03 m·s^−1^
				60%1RM	Mean velocity	CV: 9.27%; SEM: 0.02 m·s^−1^
					Mean propulsive velocity	CV: 13.81%; SEM: 0.03 m·s^−1^
					Maximum velocity	CV: 9.91%; SEM: 0.04 m·s^−1^
				70%1RM	Mean velocity	CV: 9.50%; SEM: 0.02 m·s^−1^
					Mean propulsive velocity	CV: 12.27%; SEM: 0.02 m·s^−1^
					Maximum velocity	CV: 8.68%; SEM: 0.03 m·s^−1^
				80%1RM	Mean velocity	CV: 9.09%; SEM: 0.02 m·s^−1^
					Mean propulsive velocity	CV: 9.07%; SEM: 0.02 m·s^−1^
					Maximum velocity	CV: 5.67%; SEM: 0.02 m·s^−1^
				90%1RM	Mean velocity	CV: 11.02%; SEM: 0.05 m·s^−1^
					Mean propulsive velocity	CV: 11.00%; SEM: 0.02 m·s^−1^
					Maximum velocity	CV: 7.73%; SEM: 0.02 m·s^−1^
				100%1RM	Mean velocity	CV: 16.77%; SEM: 0.02 m·s^−1^
					Mean propulsive velocity	CV: 17.26%; SEM: 0.02 m·s^−1^
					Maximum velocity	CV: 9.79%; SEM: 0.02 m·s^−1^
Garcia-Ramos et al. [71]	T-Force	Intra-device	F/W Back Squat	20 kg Load	Mean velocity	SEM: 0.052 m·s^−1^; CV: 4.65%; ICC: 0.90
					Mean propulsive velocity	SEM: 0.059 m·s^−1^; CV: 4.87%; ICC: 0.91
					Maximum velocity	SEM: 0.075 m·s^−1^; CV: 4.17%; ICC: 0.93
				40 kg Load	Mean velocity	SEM: 0.040 m·s^−1^; CV: 4.19%; ICC: 0.93
					Mean propulsive velocity	SEM: 0.046 m·s^−1^; CV: 4.46%; ICC: 0.92
					Maximum velocity	SEM: 0.058 m·s^−1^; CV: 3.59%; ICC: 0.95
				50 kg Load	Mean velocity	SEM: 0.038 m·s^−1^; CV: 4.25%; ICC: 0.90
					Mean propulsive velocity	SEM: 0.047 m·s^−1^; CV: 4.83%; ICC: 0.87
					Maximum velocity	SEM: 0.044 m·s^−1^; CV: 2.84%; ICC: 0.95
				60 kg Load	Mean velocity	SEM: 0.031 m·s^−1^; CV: 3.75%; ICC: 0.94
					Mean propulsive velocity	SEM: 0.033 m·s^−1^; CV: 3.73%; ICC: 0.95
					Maximum velocity	SEM: 0.049 m·s^−1^; CV: 3.35%; ICC: 0.94
				70 kg Load	Mean velocity	SEM: 0.036 m·s^−1^; CV: 4.84%; ICC: 0.92
					Mean propulsive velocity	SEM: 0.036 m·s^−1^; CV: 4.49%; ICC: 0.93
					Maximum velocity	SEM: 0.047 m·s^−1^; CV: 3.45%; ICC: 0.95
Garnacho-Castano et al. [56]	Tendo	Intra-device	S/M Back Squat	40–60 kg,	Mean velocity	Bias: − 0.02 ± 0.07 m·s^−1^; CV: 8.5%
					Peak velocity	Bias: − 0.05 ± 0.13 m·s^−1^; CV: 9.6%
				+ 85%1RM	Mean power	Bias: − 5.45 ± 39.75W; CV: 10.9%
					Peak power	Bias: − 19.99 ± 85.78W; CV: 13.0%
	Tendo	Intra-device	S/M Bench Press	40–60 kg,	Mean velocity	Bias: 0.001 ± 0.08 m·s^−1^; CV: 9.6%
					Peak velocity	Bias: − 0.004 ± 0.08 m·s^−1^; CV: 9.0%
				+ 85%1RM	Mean power	Bias: − 3.53 ± 27.87W; CV: 10.7%
					Peak power	Bias: − 18.40 ± 49.35W; CV: 13.2%
						*30%, 60%, 90%1RM*
Hughes et al. [52]	GymAware	Intra-device	F/W Back Squat	30–90%1RM	Mean velocity	ICC: 0.86, 0.78, 0.76; CV: 1.5%, 4.7%, 5.1%
			F/W Bench Press		Mean velocity	ICC: 0.97, 0.80, 0.85; CV: 0.6%, 2.0%, 2.4%
			F/W Prone Bench Pull		Mean velocity	ICC: 0.90, 0.92, 0.85; CV: 1.2%, 1.0%, 2.3%
			F/W OHP		Mean velocity	ICC: 0.88, 0.83, 0.85; CV: 1.2%, 2.4%, 2.5%
			S/M Back Squat	30–90%1RM	Mean velocity	ICC: 0.97, 0.80, 0.97; CV: 1.0%, 3.3%, 2.8%
			S/M Bench Press		Mean velocity	ICC: 0.98, 0.94, 0.93; CV: 0.4%, 1.4%, 1.7%
			S/M Prone Bench Pull		Mean velocity	ICC: 0.89, 0.98, 0.88; CV: 0.7%, 1.0%, 0.8%
			S/M OHP		Mean velocity	ICC: 0.82, 0.86, 0.91; CV: 2.2%, 4.6%, 2.3%
Jennings et al. [79]	FitroDyne (fitronic)	Intra-device	F/W Biceps curls	0–90%1RM	Maximum power	Limits of Agreement: 0.11 ± 13.60W; ICC: 0.97
			F/W Squat Jump	0–90%1RM	Maximum power	Limits of Agreement: − 17 ± 96W; ICC: 0.97
Lorenzetti et al. [54]	T-Force (v2.3)	Intra-device	F/W Back Squat	70%1RM	Mean velocity	*r:* 0.970; RMSE: 0.070 m·s^−1^
					Maximum velocity	*r:* 0.933; RMSE: 0.151 m·s^−1^ m·s^−1^
					Time to peak velocity	*r:* 0.985; RMSE: 0.026s
			F/W Ballistic Squat	25 kg	Mean velocity	*r:* 0.724; RMSE: 0.167 m·s^−1^
					Maximum velocity	*r:* 0.810; RMSE: 0.263 m·s^−1^
					Time to peak velocity	*r:* 0.655; RMSE: 0.045s
	Tendo (v4.1.0)	Intra-device	F/W Back Squat	70%1RM	Mean velocity	*r:* 0.963; RMSE: 0.046 m·s^−1^
					Maximum velocity	*r:* 0.932; RMSE: 0.194 m·s^−1^
					Time to peak velocity	*r: *0.985; RMSE: 0.041s
			F/W Ballistic Squat	25 kg	Mean velocity	*r:* 0.770; RMSE:* 0.157* m·s^−1^
					Maximum velocity	*r:* 0.860; RMSE: 0.135 m·s^−1^
					Time to peak velocity	*r:* 0.604; RMSE: 0.064s
	GymAware (v1.1.2)	Intra-device	F/W Back Squat	70%1RM	Mean velocity	*r:* 0.958; RMSE: 0.064 m·s^−1^
					Maximum velocity	*r:* 0.957; RMSE: 0.163 m·s^−1^
					Time to peak velocity	*r:* 0.990; RMSE: 0.042s
			F/W Ballistic Squat	25 kg	Mean velocity	*r:* 0.783; RMSE: 0.160 m·s^−1^
					Maximum velocity	*r:* 0.852; RMSE: 0.304 m·s^−1^
					Time to peak velocity	*r:* 0.701; RMSE: 0.046s
Martinez-Cava et al. 51]	T-Force	Inter-device	S/M Back Squat	25–95 kg	Peak velocity	SEM: 0.01 m·s^−1^; CV: 0.46%; ICC: 1.000; *r*: 0.9997
					Mean propulsive velocity	SEM: 0.01 m·s^−1^; CV: 0.58%; ICC: 1.000; *r*: 0.9998
					Mean velocity	SEM: <0.01 m·s^−1^; CV: 0.44%; ICC: 1.000; *r*: 0.9998
			S/M Bench Press	25–95 kg	Peak velocity	SEM: 0.01 m·s^−1^; CV: 0.45%; ICC: 1.000; *r*: 0.9998
					Mean propulsive velocity	SEM: 0.01 m·s^−1^; CV: 0.62%; ICC: 1.000; *r*: 0.9999
					Mean velocity	SEM: <0.01 m·s^−1^; CV: 0.55%; ICC: 1.000; *r*: 0.9999
	Speed4Lifts	Inter-device	S/M Back Squat	25–95 kg	Peak velocity	SEM: 0.01 m·s^−1^; CV: 0.86%; ICC: 0.999
					Mean propulsive velocity	SEM: 0.01 m·s^−1^; CV: 1.24%; ICC: 0.999
			S/M Bench Press	25–95 kg	Peak velocity	SEM: 0.02 m·s^−1^; CV: 1.54%; ICC: 1.000
					Mean propulsive velocity	SEM: 0.02 m·s^−1^; CV: 1.80%; ICC: 0.999
Muyor et al [38]	Tendo	Intra-device	S/M Back Squat	40%1RM	Mean velocity (con)	ES: 0.08; SEM: 0.007 m·s^−1^; CV: 2.00%; ICC: 0.979
					Mean velocity (ecc)	ES: 0.15; SEM: 0.009 m·s^−1^; CV: 3.65%; ICC: 0.970
				80%1RM	Mean velocity (con)	ES: 0.24; SEM: 0.013 m·s^−1^; CV: 4.28%; ICC: 0.855
					Mean velocity (ecc)	ES: 0.10; SEM: 0.011 m·s^−1^; CV: 4.55%; ICC: 0.924
Orange et al. [48]	GymAware	Intra-device	F/W Back Squat	20%1RM	Mean velocity	Standardised mean bias: 0.21; SEM: 0.05 m·s^−1^; ICC: 0.72
					Peak velocity	Standardised mean bias: 0.08; SEM: 0.09 m·s^−1^; ICC: 0.77
					Mean power	Standardised mean bias: 0.19; SEM: 102.5W; ICC: 0.79
					Peak power	Standardised mean bias: 0.04; SEM: 250.4W; ICC: 0.81
				40%1RM	Mean velocity	Standardised mean bias: 0.22; SEM: 0.04 m·s^−1^; ICC: 0.77
					Peak velocity	Standardised mean bias: 0.08; SEM: 0.07 m·s^−1^; ICC: 0.78
					Mean power	Standardised mean bias: 0.12; SEM: 79.6W; ICC: 0.82
					Peak power	Standardised mean bias: 0.02; SEM: 219.1W; ICC: 0.84
				60%1RM	Mean velocity	Standardised mean bias: 0.06; SEM: 0.04 m·s^−1^; ICC: 0.83
					Peak velocity	Standardised mean bias: 0.13; SEM: 0.06 m·s^−1^; ICC: 0.79
					Mean power	Standardised mean bias: 0.07; SEM: 73.0W; ICC: 0.81
					Peak power	Standardised mean bias: 0.04; SEM: 196.4W; ICC: 0.77
				80%1RM	Mean velocity	Standardised mean bias: 0.22; SEM: 0.03 m·s^−1^; ICC: 0.83
					Peak velocity	Standardised mean bias: 0.33; SEM: 0.06 m·s^−1^; ICC: 0.68
					Mean power	Standardised mean bias: 0.23; SEM: 76.7W; ICC: 0.79
					Peak power	Standardised mean bias: 0.43; SEM: 217.0W; ICC: 0.60
				90%1RM	Mean velocity	Standardised mean bias: 0.11; SEM: 0.04 m·s^−1^; ICC:0.79
					Peak velocity	Standardised mean bias: 0.42; SEM: 0.06 m·s^−1^; ICC: 0.65
					Mean power	Standardised mean bias: 0.20; SEM: 76.2W; ICC: 0.77
					Peak power	Standardised mean bias: 0.50; SEM: 202.7W; ICC: 0.58
			F/W Bench Press	20%1RM	Mean velocity	Standardised mean bias: 0.56; SEM: 0.09 m·s^−1^; ICC: 0.64
					Peak velocity	Standardised mean bias: 0.27; SEM: 0.13 m·s^−1^; ICC: 0.70
					Mean power	Standardised mean bias: 0.33; SEM: 52.8W; ICC: 0.81
					Peak power	Standardised mean bias: 0.14; SEM: 60.9W; ICC: 0.87
				40%1RM	Mean velocity	Standardised mean bias: 0.27; SEM: 0.05 m·s^−1^; ICC: 0.71
					Peak velocity	Standardised mean bias: 0.21; SEM: 0.06 m·s^−1^; ICC: 0.82
					Mean power	Standardised mean bias: 0.20; SEM: 27.4W; ICC: 0.91
					Peak power	Standardised mean bias: 0.16; SEM: 43.2W; ICC: 0.91
				60%1RM	Mean velocity	Standardised mean bias: 0.09; SEM: 0.04 m·s^−1^; ICC: 0.70
					Peak velocity	Standardised mean bias: 0.12; SEM: 0.05 m·s^−1^; ICC: 0.81
					Mean power	Standardised mean bias: 0.07; SEM: 27.1W; ICC: 0.89
					Peak power	Standardised mean bias: 0.16; SEM: 38.7W; ICC: 0.89
				80%1RM	Mean velocity	Standardised mean bias: 0.00; SEM: 0.04 m·s^−1^; ICC: 0.78
					Peak velocity	Standardised mean bias: 0.03; SEM: 0.06 m·s^−1^; ICC: 0.77
					Mean power	Standardised mean bias: 0.00; SEM: 28.2W; ICC: 0.83
					Peak power	Standardised mean bias: 0.06; SEM: 51.8W; ICC: 0.77
				90%1RM	Mean velocity	Standardised mean bias: 0.00; SEM: 0.03 m·s^−1^; ICC: 0.87
					Peak velocity	Standardised mean bias: 0.03; SEM: 0.07 m·s^−1^; ICC: 0.68
					Mean power	Standardised mean bias: 0.00; SEM: 29.6W; ICC: 0.85
					Peak power	Standardised mean bias: 0.06; SEM:78.0W; ICC: 0.64
Perez-Castilla et al. [10]	T-Force	Intra-device	S/M Bench Press	45%1RM	Mean velocity	CV: 2.48%; ICC: 0.90
				55%1RM	Mean velocity	CV: 1.82%; ICC: 0.95
				65%1RM	Mean velocity	CV: 4.35%; ICC: 0.78
				75%1RM	Mean velocity	CV: 4.78%; ICC: 0.77
				85%1RM	Mean velocity	CV: 4.90%; ICC: 0.87
	Chronojump	Intra-device	S/M Bench Press	45%1RM	Mean velocity	CV: 2.31%; ICC: 0.87
				55%1RM	Mean velocity	CV: 2.09%; ICC: 0.90
				65%1RM	Mean velocity	CV: 6.24%; ICC: 0.72
				75%1RM	Mean velocity	CV: 4.53%; ICC: 0.85
				85%1RM	Mean velocity	CV: 5.65%; ICC: 0.86
				45%1RM	Mean velocity	CV: 2.61%; ICC: 0.87
	Speed4Lift	Intra-device	S/M Bench Press	55%1RM	Mean velocity	CV: 2.39%; ICC: 0.84
				65%1RM	Mean velocity	CV: 2.42%; ICC: 0.93
				75%1RM	Mean velocity	CV: 3.92%; ICC: 0.81
				85%1RM	Mean velocity	CV: 3.41%; ICC: 0.94
Stock et al. [78]	Tendo	Intra-device	F/W Bench Press	10%1RM	Mean velocity	MD: 0.35 m·s^−1^; SEM: 4.2%; ICC: 0.717
				20%1RM	Mean velocity	MD: 0.33 m·s^−1^; SEM: 5.0%; ICC: 0.572
				30%1RM	Mean velocity	MD: 0.17 m·s^−1^; SEM: 3.1%; ICC: 0.805
				40%1RM	Mean velocity	MD: 0.21 m·s^−1^; SEM: 4.7%; ICC: 0.669
				50%1RM	Mean velocity	MD: 0.17 m·s^−1^; SEM: 4.6%; ICC: 0.790
				60%1RM	Mean velocity	MD: 0.15 m·s^−1^; SEM: 4.8%; ICC: 0.785
				70%1RM	Mean velocity	MD: 0.14 m·s^−1^; SEM: 5.8%; ICC: 0.811
				80%1RM	Mean velocity	MD: 0.19 m·s^−1^; SEM: 10.3%; ICC: 0.714
				90%1RM	Mean velocity	MD: 0.18 m·s^−1^; SEM: 12.6%; ICC: 0.564
Thompson et al. [24]	GymAware	Intra-device	F/W Back Squat	40%1RM	Mean velocity	TE: 0.04 m·s^−1^; CV: 4.5%
					Peak velocity	TE: 0.08 m·s^−1^; CV: 5.6%
				50%1RM	Mean velocity	TE: 0.03 m·s^−1^; CV: 3.4%
					Peak velocity	TE: 0.07 m·s^−1^; CV: 4.9%
				60%1RM	Mean velocity	TE: 0.02 m·s^−1^; CV: 2.9%
					Peak velocity	TE: 0.08 m·s^−1^; CV: 6.0%
				70%1RM	Mean velocity	TE: 0.03 m·s^−1^; CV: 4.5%
					Peak velocity	TE: 0.10 m·s^−1^; CV: 8.3%
				80%1RM	Mean velocity	TE: 0.04 m·s^−1^; CV: 7.0%
					Peak velocity	TE: 0.09 m·s^−1^; CV: 8.6%
				90%1RM	Mean velocity	TE: 0.04 m·s^−1^; CV: 9.5%
					Peak velocity	TE: 0.09 m·s^−1^; CV: 12.6%
				100%1RM	Mean velocity	TE: 0.03 m·s^−1^; CV: 13.6%
					Peak velocity	TE: 0.15 m·s^−1^; CV: 22.0%
				Full	Mean velocity	TE: 0.04 m·s^−1^; CV: 9.8%
					Peak velocity	TE: 0.10 m·s^−1^; CV: 11.3%
			Power clean	40%1RM	Mean velocity	TE: 0.05 m·s^−1^; CV: 3.6%
					Peak velocity	TE: 0.09 m·s^−1^; CV: 3.7%
				50%1RM	Mean velocity	TE: 0.03 m·s^−1^; CV: 2.2%
					Peak velocity	TE: 0.08 m·s^−1^; CV: 3.7%
				60%1RM	Mean velocity	TE: 0.03 m·s^−1^; CV: 2.4%
					Peak velocity	TE: 0.07 m·s^−1^; CV: 3.1%
				70%1RM	Mean velocity	TE: 0.04 m·s^−1^; CV: 3.2%
					Peak velocity	TE: 0.05 m·s^−1^; CV: 2.5%
				80%1RM	Mean velocity	TE: 0.04 m·s^−1^; CV: 3.3%
					Peak velocity	TE: 0.08 m·s^−1^; CV: 3.8%
				90%1RM	Mean velocity	TE: 0.08 m·s^−1^; CV: 8.9%
					Peak velocity	TE: 0.07 m·s^−1^; CV: 3.9%
				100%1RM	Mean velocity	TE: 0.04 m·s^−1^; CV: 4.3%
					Peak velocity	TE: 0.06 m·s^−1^; CV: 4.0%
				Full	Mean velocity	TE: 0.05 m·s^−1^; CV: 4.9%
					Peak velocity	TE: 0.07 m·s^−1^; CV: 3.3%
van den Tillaar and Ball [11]	Musclelab (Ergotest)	Intra-device	F/W Bench Press	50%1RM	Mean velocity	ICC: 0.98; CV: 6.6 ± 2.4%; *r: *0.96
				+ 10 kg	peak velocity	ICC: 0.98; CV: 6.9 ± 2.0%; *r:* 0.96
				+ 10 kg		
				+ 10 kg		
			F/W push-up	Body weight, 10–20–30 kg Weight Vest	mean velocity	ICC: 0.98; CV: 5.9 ± 1.7%; *r: *0.95
					peak velocity	ICC: 0.98; CV: 7.3 ± 3.0%; *r:* 0.95

*1RM* one repetition maximum, *T-Force* T-force linear velocity transducer, *GymAware* GymAware PowerTool, *Tendo* tendo weightlifting analyser, *CV* coefficient of variation, *TE* typical error, *S/M* Smith machine, *MD* mean difference, *r* Pearson’s correlation coefficient, *ICC* intraclass correlation coefficient, *SEM* standard error of measurement, *OHP* overhead press, *PBP* prone bench pull, *RMSE* root-mean-square error

**Table 9:** Summary of studies that investigated the reliability of accelerometer devices used to measure kinetic and kinematic variables during resistance training

Study	Device	Reliability	Exercise	Intensity/load	Variable measured	Findings
Abbott et al. [59]	Bar Sensei	Intra-device	F/W Back Squat	20%1RM	Peak velocity	CV: 14.17%
					Mean velocity	CV: 18.97%
					Mean propulsive velocity	CV: 15.42%
				30%1RM	Peak velocity	CV: 12.44%
					Mean velocity	CV: 15.79%
					Mean propulsive velocity	CV: 15.73%
				40%1RM	Peak velocity	CV: 13.39%
					Mean velocity	CV: 17.58%
					Mean propulsive velocity	CV: 16.13%
				50%1RM	Peak velocity	CV: 15.38%
					Mean velocity	CV: 20.89%
					Mean propulsive velocity	CV: 17.98%
				60%1RM	Peak velocity	CV: 17.86%
					Mean velocity	CV: 19.24%
					Mean propulsive velocity	CV: 20.2%
				70%1RM	Peak velocity	CV: 23.97%
					Mean velocity	CV: 18.82%
					Mean propulsive velocity	CV: 19.21%
				80%1RM	Peak velocity	CV: 31.43%
					Mean velocity	CV: 25.51%
					Mean propulsive velocity	CV: 25.71%
				90%1RM	Peak velocity	CV: 33.36%
					Mean velocity	CV: 29.94%
					Mean propulsive velocity	CV: 25.58%
				100%1RM	Peak velocity	CV: 43.77%
					Mean velocity	CV: 43.02%
					Mean propulsive velocity	CV: 34.59%
Balsalobre-Fernández	PUSH Band	Intra-device	S/M Back Squat	20–70 kg	Peak velocity	CV: 6.0 ± 3.9%; ICC: 0.981; *r: *0.952
et al. [62]					Mean velocity	CV: 5.0 ± 4.1%; ICC: 0.978; *r*: 0.956
Balsalobre-Fernández et al. [69]	Beast Sensor (Wrist)	Intra-device	F/W Back Squat	50–95%1RM	Mean velocity	ICC: 0.975
			F/W Bench Press	50–95%1RM	Mean velocity	ICC: 0.977
			F/W Hip Thrust	50–95%1RM	Mean velocity	ICC: 0.952
	Beast Sensor (Barbell)	Intra-device	F/W Back Squat	50–95%1RM	Mean velocity	ICC: 0.979
			F/W Bench Press	50–95%1RM	Mean velocity	ICC: 0.981
			F/W Hip Thrust	50–95%1RM	Mean velocity	ICC: 0.958
Beckham et al. [28]	Bar Sensei	Intra-device	F/W Back Squat	45%1RM	Mean velocity	ICC: 0.419
					Peak velocity	ICC: 0.451
				60%1RM	Mean velocity	ICC: 0.171
					Peak velocity	ICC: 0.273
				75%1RM	Mean velocity	ICC: 0.295
					Peak velocity	ICC: 0.349
Courel-Ibanez et al. [36]	Push Band	Intra-device	S/M Bench Press	20–80 kg	Mean velocity	SEM: 0.08 m·s^−1^; CV: 12.2%; ICC: 0.974
					Peak velocity	SEM: 0.18 m·s^−1^; CV: 13.7%; ICC: 0.962
			S/M Back Squat	20–80 kg	Mean velocity	SEM: 0.06 m·s^−1^; CV: 5.6%; ICC: 0.979
					Peak velocity	SEM: 0.09 m·s^−1^; CV: 5.9%; ICC: 0.944
Garcia-Pinillos et al. [61]	WIMU System	Intra-device	S/M Concentric-Only Half Back Squat	10%1RM	Mean velocity	CV: 9.02%; SEM: 0.03 m·s^−1^
					Mean propulsive velocity	CV: 11.69%; SEM: 0.04 m·s^−1^
					Maximum velocity	CV: 11.76%; SEM: 0.07 m·s^−1^
				20%1RM	Mean velocity	CV: 6.19%; SEM: 0.02 m·s^−1^
					Mean propulsive velocity	CV: 8.14%; SEM: 0.03 m·s^−1^
					Maximum velocity	CV: 8.45%; SEM: 0.05 m·s^−1^
				30%1RM	Mean velocity	CV: 11.77%; SEM: 0.11 m·s^−1^
					Mean propulsive velocity	CV: 12.44%; SEM: 0.03 m·s^−1^
					Maximum velocity	CV: 14.44%; SEM: 0.06 m·s^−1^
				40%1RM	Mean velocity	CV: 7.90%; SEM: 0.06 m·s^−1^
					Mean propulsive velocity	CV: 8.32%; SEM: 0.02 m·s^−1^
					Maximum velocity	CV: 7.48%; SEM: 0.03 m·s^−1^
				50%1RM	Mean velocity	CV: 7.86%; SEM: 0.06 m·s^−1^
					Mean propulsive velocity	CV: 8.99%; SEM: 0.02 m·s^−1^
					Maximum velocity	CV: 7.52%; SEM: 0.03 m·s^−1^
				60%1RM	Mean velocity	CV: 10.41%; SEM: 0.02 m·s^−1^
					Mean propulsive velocity	CV: 11.23%; SEM: 0.02 m·s^−1^
					Maximum velocity	CV: 13.62%; SEM: 0.05 m·s^−1^
				70%1RM	Mean velocity	CV: 13.82%; SEM: 0.02 m·s^−1^
					Mean propulsive velocity	CV: 16.75%; SEM: 0.03 m·s^−1^
					Maximum velocity	CV: 16.80%; SEM: 0.05 m·s^−1^
				80%1RM	Mean velocity	CV: 12.04%; SEM: 0.02 m·s^−1^
					Mean propulsive velocity	CV: 13.23%; SEM: 0.02 m·s^−1^
					Maximum velocity	CV: 7.18%; SEM: 0.03 m·s^−1^
				90%1RM	Mean velocity	CV: 12.62%; SEM: 0.06 m·s^−1^
					Mean propulsive velocity	CV: 16.47%; SEM: 0.02 m·s^−1^
					Maximum velocity	CV: 11.77%; SEM: 0.03 m·s^−1^
				100%1RM	Mean velocity	CV: 13.27%; SEM: 0.01 m·s^−1^
					Mean propulsive velocity	CV: 14.21%; SEM: 0.01 m·s^−1^
					Maximum velocity	CV: 15.66%; SEM: 0.03 m·s^−1^
						30%, 60%, 90%1RM
Hughes et al. [52]	PUSH Band 2.0 (arm)	Intra-device	F/W Back Squat	30–90%1RM	Mean velocity	ICC: 0.89, 0.86, 0.85; CV: 1.6%, 4.6%, 5.5%
			F/W Bench Press		Mean velocity	ICC: 0.94, 0.90, 0.88; CV: 0.8%, 1.6%, 2.5%
			F/W Prone Bench Pull		Mean velocity	ICC: 0.84, 0.88, 0.85; CV: 1.3%, 1.2%, 2.0%
			F/W OHP		Mean velocity	ICC: 0.67, 0.88, 0.75; CV: 3.3%, 3.7%, 4.2%
			S/M Back Squat	30–90%1RM	Mean velocity	ICC: 0.86, 0.94, 0.85; CV: 1.3%, 3.3%, 6.9%
			S/M Bench Press		Mean velocity	ICC: 0.83, 0.85, 0.95; CV: 1.1%, 2.4%, 4.2%
			S/M Prone Bench Pull		Mean velocity	ICC: 0.80, 0.94, 0.75; CV: 2.1%, 1.5%, 2.3%
			S/M OHP		Mean velocity	ICC: 0.80, 0.80, 0.75; CV: 2.1%, 5.1%, 3.2%
	PUSH Band 2.0 (bar)	Intra-device	F/W Back Squat	30–90%1RM	Mean velocity	ICC: 0.81, 0.80, 0.85; CV: 1.9%, 5.1%, 7.1%
			F/W Bench Press		Mean velocity	ICC: 0.84, 0.88, 0.80; CV: 1.5%, 0.7%, 3.8%
			F/W OHP		Mean velocity	ICC: 0.58, 0.75, 0.72; CV: 3.2%, 3.8%, 5.7%
			S/M Back Squat	30–90%1RM	Mean velocity	ICC: 0.87, 0.93, 0.80; CV: 1.7%, 4.6%, 6.5%
			S/M Bench Press		Mean velocity	ICC: 0.88, 0.74, 0.75; CV: 1.2%, 3.5%, 3.8%
			S/M OHP		Mean velocity	ICC: 0.86, 0.81, 0.65; CV: 1.8%, 3.0%, 4.2%
Lake et al. [29]	PUSH Band 2.0 (bar)	Intra-device	F/W Bench Press	60%1RM	Peak velocity	ICC: 0.947; CV: 4.2%
					Mean velocity	ICC: 0.937; CV: 5.8%
				90%1RM	Peak velocity	ICC: 0.957; CV: 4.7%
					Mean velocity	ICC: 0.973; CV: 7.2%
Lorenzetti et al. [54]	MyoTest	Intra-device	F/W Ballistic Squat	25 kg	Mean velocity	*r:* 0.610
					Maximum velocity	*r:* 0.552
					Time to peak velocity	*r: *0.700
Garcia-Mateo [63]	RehaGait	Inter-device	F/W Squat with arms extended	<1 kg	Mean velocity	MD ± SD: 0.046 ± 0.052 m·s^−1^
Muyor et al [38]	WIMU	Intra-device	S/M Back Squat	40%1RM	Mean velocity (con)	ES: 0.00; SEM: 0.007 m·s^−1^; CV: 2.60%; ICC: 0.976
					Mean velocity (ecc)	ES: 0.06; SEM: 0.013 m·s^−1^; CV: 3.79%; ICC: 0.955
				80%1RM	Mean velocity (con)	ES: 0.00; SEM: 0.011 m·s^−1^; CV: 3.53%; ICC: 0.905
					Mean velocity (ecc)	ES: 0.11; SEM: 0.010 m·s^−1^; CV: 4.51%; ICC: 0.924
Orange et al. [47]	PUSH Band (arm)	Intra-device	F/W Back Squat	20%1RM	Mean velocity	SEM: 0.08 m·s^−1^; ICC: 0.68
					Peak velocity	SEM: 0.12 m·s^−1^; ICC: 0.71
					Mean power	SEM: 128.3W; ICC: 0.82
					Peak power	SEM: 261.2W; ICC: 0.80
			F/W Back Squat	40%1RM	Mean velocity	SEM: 0.07 m·s^−1^; ICC:0.62
					Peak velocity	SEM: 0.18 m·s^−1^; ICC: 0.25
					Mean power	SEM: 121.5W; ICC: 0.67
					Peak power	SEM: 345.8W; ICC: 0.66
			F/W Back Squat	60%1RM	Mean velocity	SEM: 0.06 m·s^−1^; ICC: 0.64
					Peak velocity	SEM: 0.11 m·s^−1^; ICC: 0.55
					Mean power	SEM: 105.9W; ICC: 0.58
					Peak power	SEM: 279.4W; ICC: 0.67
			F/W Back Squat	80%1RM	Mean velocity	SEM: 0.06 m·s^−1^; ICC: 0.60
					Peak velocity	SEM: 0.11 m·s^−1^; ICC: 0.44
					Mean power	SEM: 129.5W; ICC: 0.37
					Peak power	SEM: 345.4W; ICC: 0.27
			F/W Back Squat	90%1RM	Mean velocity	SEM: 0.06 m·s^−1^; ICC: 0.36
					Peak velocity	SEM: 0.12 m·s^−1^; ICC: 0.66
					Mean power	SEM: 117.0W; ICC: 0.41
					Peak power	SEM: 359.5W; ICC: 0.34
	PUSH Band (arm)	Intra-device	F/W Bench Press	20%1RM	Mean velocity	SEM: 0.11 m·s^−1^; ICC: 0.28
					Peak velocity	SEM: 0.21 m·s^−1^; ICC: 0.27
					Mean power	SEM: 70.6W; ICC: 0.43
					Peak power	SEM: 221.9W; ICC: 0.63
			F/W Bench Press	40%1RM	Mean velocity	SEM: 0.08 m·s^−1^; ICC: 0.60
					Peak velocity	SEM: 0.11 m·s^−1^; ICC: 0.66
					Mean power	SEM: 33.8W; ICC: 0.83
					Peak power	SEM: 151.0W; ICC: 0.89
			F/W Bench Press	60%1RM	Mean velocity	SEM: 0.08 m·s^−1^; ICC: 0.58
					Peak velocity	SEM: 0.12 m·s^−1^; ICC:58
					Mean power	SEM: 51.6W; ICC: 0.69
					Peak power	SEM: 273.0W; ICC: 0.38
			F/W Bench Press	80%1RM	Mean velocity	SEM: 0.06 m·s^−1^; ICC: 0.51
					Peak velocity	SEM: 0.08 m·s^−1^; ICC: 0.47
					Mean power	SEM: 51.3W; ICC: 0.58
					Peak power	SEM: 137.5W; ICC: 0.55
			F/W Bench Press	90%1RM	Mean velocity	SEM: 0.05 m·s^−1^; ICC: 0.37
					Peak velocity	SEM: 0.10 m·s^−1^; ICC: 0.40
					Mean power	SEM: 45.7W; ICC: 0.67
					Peak power	SEM: 131.9W; ICC: 0.59
Perez-Castilla et al. [10]	PUSH Band (arm)	Intra-device	S/M Bench Press	45%1RM	Mean velocity	CV: 5.02%; ICC: 0.69
				55%1RM	Mean velocity	CV: 7.84%; ICC: 0.46
				65%1RM	Mean velocity	CV: 9.34%; ICC: 0.78
				75%1RM	Mean velocity	CV: 14.6%; ICC: 0.50
	Beast Sensor			85%1RM	Mean velocity	CV: 19.1%; ICC: 0.47
		Intra-device	S/M Bench Press	45%1RM	Mean velocity	CV: 33.4%; ICC: 0.29
				55%1RM	Mean velocity	CV: 24.2%; ICC: 0.64
				65%1RM	Mean velocity	CV: 35.0%; ICC: 0.30
				75%1RM	Mean velocity	CV: 40.2%; ICC: 0.31
				85%1RM	Mean velocity	CV: 54.9%; ICC: 0.27
Thompson et al. [24]	PUSH Band (body)	Intra-device	F/W Back Squat	40%1RM	Mean velocity	TE: 0.04 m·s^−1^; CV: 3.5%
					Peak velocity	TE: 0.08 m·s^−1^; CV: 6.0%
				50%1RM	Mean velocity	TE: 0.03 m·s^−1^; CV: 4.1%
					Peak velocity	TE: 0.07 m·s^−1^; CV: 9.9%
				60%1RM	Mean velocity	TE: 0.02 m·s^−1^; CV: 5.4%
					Peak velocity	TE: 0.08 m·s^−1^; CV: 9.1%
				70%1RM	Mean velocity	TE: 0.03 m·s^−1^; CV: 5.0%
					Peak velocity	TE: 0.10 m·s^−1^; CV: 8.9%
				80%1RM	Mean velocity	TE: 0.04 m·s^−1^; CV: 5.2%
					Peak velocity	TE: 0.09 m·s^−1^; CV: 6.8%
				90%1RM	Mean velocity	TE: 0.04 m·s^−1^; CV: 15.6%
					Peak velocity	TE: 0.09 m·s^−1^; CV: 11.0%
				100%1RM	Mean velocity	TE: 0.03 m·s^−1^; CV: 14.9%
					Peak velocity	TE: 0.15 m·s^−1^; CV: 11.4%
				Full	Mean velocity	TE: 0.04 m·s^−1^; CV: 10.6%
					Peak velocity	TE: 0.10 m·s^−1^; CV: 11.3%
			Power Clean	40%1RM	Mean velocity	TE: 0.06 m·s^−1^; CV: 4.9%
					Peak velocity	TE: 0.08 m·s^−1^; CV: 4.9%
				50%1RM	Mean velocity	TE: 0.06 m·s^−1^; CV: 5.2%
					Peak velocity	TE: 0.08 m·s^−1^; CV: 5.2%
				60%1RM	Mean velocity	TE: 0.05 m·s^−1^; CV: 4.5%
					Peak velocity	TE: 0.08 m·s^−1^; CV: 4.5%
				70%1RM	Mean velocity	TE: 0.09 m·s^−1^; CV: 7.7%
					Peak velocity	TE: 0.12 m·s^−1^; CV: 7.7%
				80%1RM	Mean velocity	TE: 0.10 m·s^−1^; CV: 10.2%
					Peak velocity	TE: 0.14 m·s^−1^; CV: 10.2%
				90%1RM	Mean velocity	TE: 0.09 m·s^−1^; CV: 11.3%
					Peak velocity	TE: 0.13 m·s^−1^; CV: 11.3%
				100%1RM	Mean velocity	TE: 0.09 m·s^−1^; CV: 11.4%
					Peak velocity	TE: 0.12 m·s^−1^; CV: 11.4%
				Full	Mean velocity	TE: 0.08 m·s^−1^; CV: 8.3%
					Peak velocity	TE: 0.11 m·s^−1^; CV: 8.3%
	PUSH Band (bar)	Intra-device	F/W Back Squat	40%1RM	Mean velocity	TE: 0.06 m·s^−1^; CV: 5.2%
					Peak velocity	TE: 0.09 m·s^−1^; CV: 5.7%
				50%1RM	Mean velocity	TE: 0.08 m·s^−1^; CV: 9.2%
					Peak velocity	TE: 0.10 m·s^−1^; CV: 7.5%
				60%1RM	Mean velocity	TE: 0.05 m·s^−1^; CV: 5.1%
					Peak velocity	TE: 0.12 m·s^−1^; CV: 9.4%
				70%1RM	Mean velocity	TE: 0.04 m·s^−1^; CV: 5.9%
					Peak velocity	TE: 0.09 m·s^−1^; CV: 8.3%
				80%1RM	Mean velocity	TE: 0.09 m·s^−1^; CV: 14.3%
					Peak velocity	TE: 0.09 m·s^−1^; CV: 8.8%
				90%1RM	Mean velocity	TE: 0.09 m·s^−1^; CV: 20.3%
					Peak velocity	TE: 0.12 m·s^−1^; CV: 14.2%
				100%1RM	Mean velocity	TE: 0.06 m·s^−1^; CV: 15.4%
					Peak velocity	TE: 0.09 m·s^−1^; CV: 11.6%
				Full	Mean velocity	TE: 0.07 m·s^−1^; CV: 14.5%
					Peak velocity	TE: 0.11 m·s^−1^; CV: 11.0%
			Power Clean	40%1RM	Mean velocity	TE: 0.20 m·s^−1^; CV: 21.5%
					Peak velocity	TE: 0.36 m·s^−1^; CV: 21.5%
				50%1RM	Mean velocity	TE: 0.18 m·s^−1^; CV: 19.0%
					Peak velocity	TE: 0.33 m·s^−1^; CV: 17.9%
				60%1RM	Mean velocity	TE: 0.17 m·s^−1^; CV: 18.9%
					Peak velocity	TE: 0.42 m·s^−1^; CV: 25.4%
				70%1RM	Mean velocity	TE: 0.13 m·s^−1^; CV: 14.6%
					Peak velocity	TE: 0.22 m·s^−1^; CV: 13.4%
				80%1RM	Mean velocity	TE: 0.14 m·s^−1^; CV: 16.3%
					Peak velocity	TE: 0.25 m·s^−1^; CV: 15.6%
				90%1RM	Mean velocity	TE: 0.15 m·s^−1^; CV: 18.1%
					Peak velocity	TE: 0.31 m·s^−1^; CV: 22.2%
				100%1RM	Mean velocity	TE: 0.10 m·s^−1^; CV: 13.3%
					Peak velocity	TE: 0.23 m·s^−1^; CV: 17.5%
				Full	Mean velocity	TE: 0.21 m·s^−1^; CV: 18.6%
					Peak velocity	TE: 0.32 m·s^−1^; CV: 20.5%
	Bar Sensei	Intra-device	F/W Back Squat	40%1RM	Mean velocity	TE: 0.08 m·s^−1^; CV: 9.1%
					Peak velocity	TE: 0.14 m·s^−1^; CV: 9.4%
				50%1RM	Mean velocity	TE: 0.09 m·s^−1^; CV: 13.5%
					Peak velocity	TE: 0.10 m·s^−1^; CV: 7.6%
				60%1RM	Mean velocity	TE: 0.07 m·s^−1^; CV: 8.8%
					Peak velocity	TE: 0.08 m·s^−1^; CV: 8.0%
				70%1RM	Mean velocity	TE: 0.07 m·s^−1^; CV: 10.7% TE: 0.07 m·s^−1^; CV: 10.7%
					Peak velocity	TE: 0.09 m·s^−1^; CV: 10.2%
				80%1RM	Mean velocity	TE: 0.08 m·s^−1^; CV: 18.3%
					Peak velocity	TE: 0.24 m·s^−1^; CV: 35.8%
				90%1RM	Mean velocity	TE: 0.08 m·s^−1^; CV: 19.1%
					Peak velocity	TE: 0.12 m·s^−1^; CV: 18.0%
				100%1RM	Mean velocity	TE: 0.13 m·s^−1^; CV: 60.5%
					Peak velocity	TE: 0.12 m·s^−1^; CV: 28.5%
				Full	Mean velocity	TE: 0.09 m·s^−1^; CV: 22.1%
					Peak velocity	TE: 0.13 m·s^−1^; CV: 18.7%
			Power Clean	40%1RM	Mean velocity	TE: 0.23 m·s^−1^; CV: 20.4%
					Peak velocity	TE: 0.20 m·s^−1^; CV: 7.7%
				50%1RM	Mean velocity	TE: 0.16 m·s^−1^; CV: 13.8%
					Peak velocity	TE: 0.15 m·s^−1^; CV: 6.5%
				60%1RM	Mean velocity	TE: 0.13 m·s^−1^; CV: 12.1%
					Peak velocity	TE: 0.13 m·s^−1^; CV: 5.8%
				70%1RM	Mean velocity	TE: 0.13 m·s^−1^; CV: 11.8%
					Peak velocity	TE: 0.19 m·s^−1^; CV: 8.8% TE: 0.19 m·s^−1^; CV: 8.8%
				80%1RM	Mean velocity	TE: 0.13 m·s^−1^; CV: 14.9%
					Peak velocity	TE: 0.13 m·s^−1^; CV: 6.1%
				90%1RM	Mean velocity	TE: 0.15 m·s^−1^; CV: 17.7%
					Peak velocity	TE: 0.14 m·s^−1^; CV: 7.9%
				100%1RM	Mean velocity	TE: 0.14 m·s^−1^; CV: 18.4%
					Peak velocity	TE: 0.15 m·s^−1^; CV: 8.5%
				Full	Mean velocity	TE: 0.16 m·s^−1^; CV: 15.9%
					Peak velocity	TE: 0.17 m·s^−1^; CV: 8.7%
	Beast Sensor	Intra-device	F/W Back Squat	40%1RM	Mean velocity	TE: 0.05 m·s^−1^; CV: 20.4%
					Peak velocity	TE: 0.10 m·s^−1^; CV: 7.7%
				50%1RM	Mean velocity	TE: 0.06 m·s^−1^; CV: 13.8%
					Peak velocity	TE: 0.11 m·s^−1^; CV: 6.5%
				60%1RM	Mean velocity	TE: 0.08 m·s^−1^; CV: 12.1%
					Peak velocity	TE: 0.15 m·s^−1^; CV: 5.8%
				70%1RM	Mean velocity	TE: 0.12 m·s^−1^; CV: 11.8%
					Peak velocity	TE: 0.27 m·s^−1^; CV: 8.8%
				80%1RM	Mean velocity	TE: 0.22 m·s^−1^; CV: 14.9%
					Peak velocity	TE: 0.33 m·s^−1^; CV: 6.1%
				90%1RM	Mean velocity	TE: 0.21 m·s^−1^; CV: 17.7%
					Peak velocity	TE: 0.48 m·s^−1^; CV: 7.9%
				100%1RM	Mean velocity	TE: 0.15 m·s^−1^; CV: 18.4%
					Peak velocity	TE: 0.29 m·s^−1^; CV: 8.5%
				Full	Mean velocity	TE: 0.14 m·s^−1^; CV: 15.9%
					Peak velocity	TE: 0.30 m·s^−1^; CV: 8.7%
van den Tillaar and Ball [11]	PUSH Band (arm)	Intra-device	F/W Bench Press	50%1RM	Mean velocity	ICC: 0.95; CV: 12.8 ± 2.4%; *r: *0.87
				+10 kg	Peak velocity	ICC: 0.92; CV: 13.3 ± 2.3%; *r:* 0.81
				+10 kg		
				+10 kg		
			F/W push-up	Body Weight, 10–20–30 kg Weight Vest	Mean velocity	ICC: 0.98; CV: 6.6 ± 1.3%; *r: *0.95
					Peak velocity	ICC: 0.98; CV: 6.6 ± 1.3%; *r:* 0.94

*1RM* one repetition maximum, *MV* mean concentric velocity, *CV* coefficient of variation, *S/M* Smith machine, *r* Pearson’s correlation coefficient, *ICC* intraclass correlation coefficient, *SEM* standard error of measurement, *OHP* overhead press, *RMSE* root-mean-square error

**Table 10:** Summary of studies that investigated the reliability of mobile phone and tablet applications used to measure kinetic and kinematic variables during resistance training

Study	Device	Reliability	Exercise	Intensity/load	Variable measured	Findings
Balsalobre-Fernández et al. [69]	PowerLift (v4.0)	Intra-device	F/W Back Squat	50–95%1RM	Mean velocity	ICC: 0.981
			F/W Bench Press	50–95%1RM	Mean velocity	ICC: 0.974
			F/W Hip Thrust	50–95%1RM	Mean velocity	ICC: 0.961
Balsalobre-Fernández et al. [70]	PowerLift (v2.8)	Intra-observer	F/W Bench Press	75–100%1RM	Mean velocity	MD: − 0.0007 ± 0.02 m·s^−1^
Courel-Ibanez et al. [36]	PowerLift App (v4.0)	Inter-device	S/M Bench Press	20–80 kg	Mean velocity	SEM: 0.08 m·s^−1^; CV: 10.4%; ICC: 0.973
			S/M Back Squat	20–80 kg	Mean velocity	SEM: 0.05 m·s^−1^; CV: 6.0%; ICC: 0.974
	PowerLift App (v4.0)	Intra-device	S/M Bench Press	20–80 kg	Mean velocity	SEM: 0.05 m·s^−1^; CV: 6.7%; ICC: 0.988
			S/M Back Squat	20–80 kg	Mean velocity	SEM: 0.04 m·s^−1^; CV: 4.6%; ICC: 0.986
M artinez-Cava et al. [51]	My Lift (v8.1 iOS)	Inter-device	S/M Back Squat	25–95 kg	Peak velocity	SEM: 0.08 m·s^−1^; CV: 5.79%; ICC: 0.993
			S/M Bench Press	25–95 kg	Peak velocity	SEM: 0.08 m·s^−1^; CV: 5.02%; ICC: 0.972
Perez-Castilla et al. [10]	PowerLift (v6.0.1)	Intra-device	S/M Bench Press	45%1RM	Mean velocity	CV: 2.85%; ICC: 0.84
				55%1RM	Mean velocity	CV: 3.97%; ICC: 0.85
				65%1RM	Mean velocity	CV: 4.91%; ICC: 0.74
				75%1RM	Mean velocity	CV: 3.69%; ICC: 0.87
				85%1RM	Mean velocity	CV: 4.97%; ICC: 0.85
Thompson et al. [24]	MyLift (PowerLift at time of data collection)	Intra-device	F/W Back Squat	40%1RM	Mean velocity	TE: 0.04 m·s^−1^; CV: 4.2%
				50%1RM	Mean velocity	TE: 0.03 m·s^−1^; CV: 3.7%
				60%1RM	Mean velocity	TE: 0.04 m·s^−1^; CV: 5.5%
				70%1RM	Mean velocity	TE: 0.03 m·s^−1^; CV: 4.9%
				80%1RM	Mean velocity	TE: 0.04 m·s^−1^; CV: 6.8%
				90%1RM	Mean velocity	TE: 0.05 m·s^−1^; CV: 12.6%
				100%1RM	Mean velocity	TE: 0.03 m·s^−1^; CV: 13.8%
				Full	Mean velocity	TE: 0.05 m·s^−1^; CV: 9.7%

*1RM* one repetition maximum, *CV* coefficient of variation, *S/M* Smith machine, *MD* mean difference, *ICC* intraclass correlation coefficient, *SEM* standard error of measurement, *TE* typical error

**Table 11:** Summary of studies that investigated the reliability of optic devices used to measure kinetic and kinematic variables during resistance training

Study	Device	Reliability	Exercise	Intensity/load	Variable measured	Findings
Courel-Ibanez et al. [36]	Velowin	Inter-device	S/M Bench Press	20–80 kg	Mean velocity	SEM: 0.03 m·s^−1^; CV: 3.5%; ICC: 0.997
					Mean propulsive velocity	SEM: 0.03 m·s^−1^; CV: 3.4%; ICC: 0.997
					Peak velocity	SEM: 0.03 m·s^−1^; CV: 2.1%; ICC: 0.999
			S/M Prone Bench Pull	20–80 kg	Mean velocity	SEM: 0.04 m·s^−1^; CV: 3.6%; ICC: 0.995
					Mean propulsive velocity	SEM: 0.04 m·s^−1^; CV: 3.5%; ICC: 0.995
					Peak velocity	SEM: 0.06 m·s^−1^; CV: 3.2%; ICC: 0.998
	Velowin	Intra-device	S/M Bench Press	20–80 kg	Mean velocity	SEM: 0.04 m·s^−1^; CV: 4.0%; ICC: 0.997
					Mean propulsive velocity	SEM: 0.03 m·s^−1^; CV: 3.2%; ICC: 0.998
					Peak velocity	SEM: 0.04 m·s^−1^; CV: 2.6%; ICC: 0.998
			S/M Back Squat	20–80 kg	Mean velocity	SEM: 0.04 m·s^−1^; CV: 3.7%; ICC: 0.988
					Mean propulsive velocity	SEM: 0.06 m·s^−1^; CV: 4.6%; ICC: 0.987
					Peak velocity	SEM: 0.07 m·s^−1^; CV: 3.5%; ICC: 0.983
			S/M Prone Bench Pull	20–80 kg	Mean velocity	SEM: 0.06 m·s^−1^; CV: 3.9%; ICC: 0.994
					Mean propulsive velocity	SEM: 0.06 m·s^−1^; CV: 3.9%; ICC: 0.994
					Peak velocity	SEM: 0.06 m·s^−1^; CV: 2.6%; ICC: 0.998
Peña Garcia-Orea et al. [82]	Velowin (v.1.7.232)	Intra-device	S/M Squat	20 kg	Mean velocity	ICC: 0.95; CV: 3.35%; SEM: 0.225 m·s^−1^
					Mean propulsive velocity	ICC: 0.96; CV: 3.29%; SEM: 0.287 m·s^−1^
					Peak velocity	ICC: 0.95; CV: 2.89%; SEM: 0.399 m·s^−1^
				30 kg	Mean velocity	ICC: 0.97; CV: 2.20%; SEM: 0.227 m·s^−1^
					Mean propulsive velocity	ICC: 0.97; CV: 2.45%; SEM: 0.284 m·s^−1^
					Peak velocity	ICC: 0.97; CV: 2.46%; SEM: 0.373 m·s^−1^
				40 kg	Mean velocity	ICC: 0.99; CV: 2.13%; SEM: 0.248 m·s^−1^
					Mean propulsive velocity	ICC: 0.99; CV: 2.30%; SEM: 0.306 m·s^−1^
					Peak velocity	ICC: 0.98; CV: 2.29%; SEM: 0.363 m·s^−1^
				50 kg	Mean velocity	ICC: 0.98; CV: 2.82%; SEM: 0.276 m·s^−1^
					Mean propulsive velocity	ICC: 0.99; CV: 2.98%; SEM: 0.337 m·s^−1^
					Peak velocity	ICC: 0.98; CV: 2.56%; SEM: 0.362 m·s^−1^
				60 kg	Mean velocity	ICC: 0.99; CV: 2.46%; SEM: 0.272 m·s^−1^
					Mean propulsive velocity	ICC: 0.99; CV: 2.62%; SEM: 0.316 m·s^−1^
					Peak velocity	ICC: 0.98; CV: 2.39%; SEM: 0.313 m·s^−1^
				70 kg	Mean velocity	ICC: 0.99; CV: 2.55%; SEM: 0.243 m·s^−1^
					Mean propulsive velocity	ICC: 0.99; CV: 2.79%; SEM: 0.280 m·s^−1^
					Peak velocity	ICC: 0.98; CV: 2.30%; SEM: 0.269 m·s^−1^
Peña Garcia-Orea et al. [72]	Velowin (v1.7.232)	Intra-device	S/M Loaded Countermovement Jump	3.5 kg	Mean velocity	ICC: 0.98; CV: 2.41%; SEM: 0.0025 m·s^−1^
					Peak velocity	ICC: 0.98; CV: 1.77%; SEM: 0.0021 m·s^−1^
				13.5 kg	Mean velocity	ICC: 0.97; CV: 1.70%; SEM: 0.0021 m·s^−1^
					Peak velocity	ICC: 0.99; CV: 1.68%; SEM: 0.0014 m·s^−1^
				23.5 kg	Mean velocity	ICC: 0.95; CV: 2.56%; SEM: 0.0033 m·s^−1^
					Peak velocity	ICC: 0.97; CV: 2.38%; SEM: 0.0023 m·s^−1^
				33.5 kg	Mean velocity	ICC: 0.98; CV: 1.87%; SEM: 0.0022 m·s^−1^
					Peak velocity	ICC: 0.99; CV: 1.60%; SEM: 0.0018 m·s^−1^
				43.5 kg	Mean velocity	ICC: 0.99; CV: 2.03%; SEM: 0.0040 m·s^−1^
					Peak velocity	ICC: 0.99; CV: 1.57%; SEM: 0.0027 m·s^−1^
Garcia-Ramos et al. [71]	Velowin	Intra-device	F/W Back Squat	20 kg	Mean velocity	SEM: 0.045 m·s^−1^; CV: 4.29%; ICC: 0.91
					Mean propulsive velocity	SEM: 0.054 m·s^−1^; CV: 4.61%; ICC: 0.90
					Maximum velocity	SEM: 0.088 m·s^−1^; CV: 4.77%; ICC: 0.92
			F/W Back Squat	40 kg	Mean velocity	SEM: 0.041 m·s^−1^; CV: 4.34%; ICC: 0.92
					Mean propulsive velocity	SEM: 0.047 m·s^−1^; CV: 4.60%; ICC: 0.91
					Maximum velocity	SEM: 0.085 m·s^−1^; CV: 5.01%; ICC: 0.91
			F/W Back Squat	50 kg	Mean velocity	SEM: 0.033 m·s^−1^; CV: 3.74%; ICC: 0.90
					Mean propulsive velocity	SEM: 0.043 m·s^−1^; CV: 4.50%; ICC: 0.88
					Maximum velocity	SEM: 0.050 m·s^−1^; CV: 3.04%; ICC: 0.95
			F/W Back Squat	60 kg	Mean velocity	SEM: 0.039 m·s^−1^; CV: 4.75%; ICC: 0.89
					Mean propulsive velocity	SEM: 0.037 m·s^−1^; CV: 4.20%; ICC: 0.92
					Maximum velocity	SEM: 0.069 m·s^−1^; CV: 4.44%; ICC: 0.91
			F/W Back Squat	70 kg	Mean velocity	SEM: 0.031 m·s^−1^; CV: 4.12%; ICC: 0.93
					Mean propulsive velocity	SEM: 0.041 m·s^−1^; CV: 5.15%; ICC: 0.90
					Maximum velocity	SEM: 0.053 m·s^−1^; CV: 3.57%; ICC: 0.95
Perez-Castilla et al. [10]	Velowin	Intra-device	S/M Bench Press	45%1RM	Mean velocity	CV: 2.89%; ICC: 0.83
				55%1RM	Mean velocity	CV: 3.27%; ICC: 0.79
				65%1RM	Mean velocity	CV: 3.99%; ICC: 0.83
				75%1RM	Mean velocity	CV: 6.01%; ICC: 0.68
				85%1RM	Mean velocity	CV: 7.64%; ICC: 0.69
Weakley et al. [22]	FLEX (technological and biological error)	Inter-device	F/W Back Squat	20–100%1RM	Mean velocity	MD: 0.00 m·s^−1^; TE: 0.070 m·s^−1^; CV: 9.82%
	FLEX (technological error)		F/W Bench Press	20–100%1RM	Mean velocity	MD: 0.01 m·s^−1^; TE: 0.064 m·s^−1^; CV: 9.83%
	FLEX	Inter-device	Calibrated rig	0.53 ± 0.27 m·s^−1^	Mean velocity	MD: 0.00 m·s^−1^; TE: 0.017 m·s^−1^; CV: 3.96%
				0.99 ± 0.00 m·s^−1^	Mean velocity	MD: 0.01 m·s^−1^ m·s^−1^; TE: 0.041 m·s^−1^; CV: 4.17%
				0.84 ± 0.00 m·s^−1^	Mean velocity	MD: − 0.01 m·s^−1^; TE: 0.06 m·s^−1^; CV: 7.10%
				0.78 ± 0.00 m·s^−1^	Mean velocity	MD: 0.00 m·s^−1^; TE: 0.019 m·s^−1^; CV: 2.41%
				0.71 ± 0.00 m·s^−1^	Mean velocity	MD: 0.00 m·s^−1^; TE: 0.016 m·s^−1^; CV: 2.28%
				0.60 ± 0.00 m·s^−1^	Mean velocity	MD: − 0.01 m·s^−1^; TE: 0.02 m·s^−1^; CV: 3.37%
				0.54 ± 0.00 m·s^−1^	Mean velocity	MD: 0.00 m·s^−1^; TE: 0.016 m·s^−1^; CV: 2.99%
				0.47 ± 0.00 m·s^−1^	Mean velocity	MD: 0.00 m·s^−1^; TE: 0.013 m·s^−1^; CV: 2.71%
				0.38 ± 0.00 m·s^−1^	Mean velocity	MD: 0.00 m·s^−1^; TE: 0.013 m·s^−1^; CV: 3.49%
				0.28 ± 0.00 m·s^−1^	Mean velocity	MD: 0.00 m·s^−1^; TE: 0.016 m·s^−1^; CV: 5.73%
				0.17 ± 0.00 m·s^−1^	Mean velocity	MD: 0.00 m·s^−1^; TE: 0.006 m·s^−1^; CV: 3.81%
				0.09 ± 0.00 m·s^−1^	Mean velocity	MD: 0.00 m·s^−1^; TE: 0.002 m·s^−1^; CV: 2.43%
		Intra-device	Calibrated rig	0.53 ± 0.27 m·s^−1^	Mean velocity	MD: 0.00 m·s^−1^; TE: 0.016 m·s^−1^; CV: 3.77%
				0.99 ± 0.00 m·s^−1^	Mean velocity	MD: 0.00 m·s^−1^; TE: 0.032 m·s^−1^; CV: 3.28%
				0.84 ± 0.00 m·s^−1^	Mean velocity	MD: 0.01 m·s^−1^; TE: 0.043 m·s^−1^; CV: 5.11%
				0.78 ± 0.00 m·s^−1^	Mean velocity	MD: 0.00 m·s^−1^; TE: 0.021 m·s^−1^; CV: 2.71%
				0.71 ± 0.00 m·s^−1^	Mean velocity	MD: − 0.01 m·s^−1^; TE: 0.020 m·s^−1^; CV: 2.81%
				0.60 ± 0.00 m·s^−1^	Mean velocity	MD: 0.00 m·s^−1^; TE: 0.023 m·s^−1^; CV: 3.82%
				0.54 ± 0.00 m·s^−1^	Mean velocity	MD: 0.00 m·s^−1^; TE: 0.017 m·s^−1^; CV: 3.19%
				0.47 ± 0.00 m·s^−1^	Mean velocity	MD: 0.00 m·s^−1^; TE: 0.014 m·s^−1^; CV: 3.01%
				0.38 ± 0.00 m·s^−1^	Mean velocity	MD: 0.00 m·s^−1^; TE: 0.013 m·s^−1^; CV: 3.42%
				0.28 ± 0.00 m·s^−1^	Mean velocity	MD: 0.00 m·s^−1^; TE: 0.016 m·s^−1^; CV: 5.93%
				0.17 ± 0.00 m·s^−1^	Mean velocity	MD: 0.00 m·s^−1^; TE: 0.006 m·s^−1^; CV: 3.64%
				0.09 ± 0.00 m·s^−1^	Mean velocity	MD: 0.00 m·s^−1^; TE: 0.003 m·s^−1^; CV: 2.89%

*1RM* one repetition maximum, *CV* coefficient of variation, *TE* typical error, *S/M* Smith machine, *MD* mean difference, *ICC* intraclass correlation coefficient, *SEM* standard error of measurement

Of the 19 studies that assessed the validity of linear transducer devices, 11 used free-weight equipment, six used a Smith machine, and one used both free-weight and Smith machine exercises. Relative loads from 20 to 100% of 1RM were used, while absolute loads were used within seven studies (refer to Table 4). Of the 23 studies that assessed the validity of accelerometer devices, 14 used free-weight equipment, eight used a Smith machine, and one used both free-weight and Smith machine exercises. Relative loads from 10 to 100% of 1RM were assessed, while absolute loads were used within six studies (refer to Table 5). In the 10 studies that assessed the validity of mobile phone and tablet applications, three used free-weight equipment and seven used a Smith machine. Relative loads ranging from 40 to 100% of 1RM were used, while six studies used either repetitions above or below a given speed (i.e., 0.80 m·s^−1^), absolute, or maximal (i.e., 10RM) prescriptive methods (refer to Table 6). Finally, in the eight studies that quantified the validity of optic devices, four used free-weight equipment and four used a Smith machine. Relative loads from 20 to 100% of 1RM were assessed, and one study prescribed loads at or above a given speed, while absolute loads were used within five studies (refer to Table 7).

Of the 19 studies that quantified the reliability of linear transducer devices, 10 used free-weight equipment, eight used a Smith machine, and one used both free-weight and Smith machine exercises. Relative loads from 0 to 100% of 1RM were assessed, while absolute loads were used within seven studies (refer to Table 8). Of the 14 studies that quantified the reliability of accelerometer devices, eight used free-weight equipment, five used a Smith machine, and one used both free-weight and Smith machine exercises. Relative loads from 10 to 100% of 1RM were assessed, while absolute loads were used within five studies (refer to Table 9). In the six studies that quantified the reliability of mobile phone and tablet applications, three used free-weight equipment and three used a Smith machine. Relative loads ranging from 45 to 95% of 1RM were assessed, one study used repetitions above or below a given speed, while absolute loads were used within one study (refer to Table 10). Finally, in the six studies that quantified the reliability of optic devices, two used free-weight equipment and four used a Smith machine. Relative loads from 20 to 100% of 1RM were assessed, while absolute loads were used within four studies (refer to Table 11).

## Discussion

The aims of this review were to (1) establish the level of evidence for the validity of all commercially available portable resistance training devices that monitor force, velocity, and power outputs; and, (2) determine the intra-device and inter-device reliability of these devices. Velocity was the most investigated output, with all but two studies investigating this outcome measure [49, 50]. Furthermore, it was found that most studies within this review did not utilise a gold-standard criterion measure (e.g., high-speed motion-capture set-up for measuring velocity) when assessing the validity of devices. This has likely led to under or overreporting of error for certain devices and may explain (at least in part) the inconsistent findings presented in different studies that have assessed the same device. However, when compared to a gold-standard criterion, it appears that linear transducers demonstrate greater accuracy and precision over other devices when measuring kinetic and kinematic outputs. In stating this, future research must consider utilising a broader range of exercises (e.g., Olympic weightlifting exercises and their derivatives) and loads to be confident of the reliability and validity of devices. For the assessment of reliability, only three studies have assessed the agreement between two different devices of the same brand (i.e., inter-device) [22, 36, 51]. In contrast, there has been a substantial amount of research concerning intra-device reliability [28, 48, 52]; however, it must be noted that all but one of these studies [22] failed to differentiate technological variation from biological variation to establish their respective influence on the unit’s reliability. Therefore, future research must attempt to separate these different forms of error to provide a fair reflection of the intra-device reliability and the variation that can be expected.

### Validity

Of the 19 studies that have assessed the validity of linear transducers, 10 utilised a gold-standard criterion of high-speed 3D motion capture when assessing velocity [10, 24–27, 51, 53, 54] or force plate when assessing force [8, 25, 26, 50] (Table 4). From the evidence provided, these types of devices tend to demonstrate greater accuracy when compared to accelerometers [8, 9, 26]. Of all linear transducer devices, the GymAware PowerTool [8, 9, 24–27, 50, 54, 55] and Tendo Fitrodyne [36, 53, 54, 56–58] have been the most investigated, with nine and six independent validity studies, respectively. Additionally, the Fitrodyne (Fitronic) [9, 55] and Open Barbell System [53, 57] have both had two studies assessing their validity. However, when comparing the agreement of these devices [55], it appears that there are slight discrepancies. Mitter et al. [9] demonstrated greater accuracy of the GymAware PowerTool compared to the Fitrodyne (Fitronic), while Fernandes et al. [55] warned practitioners against interchanging these devices due to systematic differences (refer to Table 4). This is particularly pertinent when utilising peak velocity [55]. Differences between devices may be due to different sampling methods (e.g., through displacement or variable rate sampling), and/or the way in which the raw data signals are treated within the software (e.g., manufacturer-defined filtering routines). Thus, while linear transducers consistently demonstrate superior accuracy compared to other forms of velocity measuring devices [8–10, 53, 54], practitioners should avoid the interchangeable use of different devices during the long-term monitoring of athletes.

Ten studies have directly compared accelerometer-based devices (i.e., Push Band versions 1.0 and 2.0, Beast sensor, BarSensei, and Myotest) to gold-standard 3D motion capture [9, 10, 24, 29, 37, 54, 58, 59] or force plate (when assessing force variables) [8, 49], with the power clean [24], bench press [9, 10, 29, 58], back squat [9, 24, 59], deadlift [9], ballistic squat [54], shoulder press [37], and the biceps curl exercise [37] being assessed (Table 5). Across these studies, all outputs, except peak velocity at 60 and 90% of 1RM in the bench press for the Push Band 2.0 [29], have demonstrated questionable validity. Furthermore, there have been additional 13 studies that have assessed accelerometer-based devices against other devices, predominantly the GymAware PowerTool [28, 47, 52, 60] or the T-Force [36, 38, 61, 62] linear transducers. From this, mean and peak velocity are the most investigated outputs. The CV from these accelerometer devices tends to range from 10 to 20% across exercises, with lighter relative loads (i.e., faster velocities) tending to have less error [8, 47]. Furthermore, monitoring mean velocity with heavy loads (i.e., > 90% of 1RM) may be extremely inaccurate (i.e., CV = 27–35%) which may be related to the detection of different phases of each movement [8, 47, 60, 63]. This may be an issue for practitioners as mean concentric velocity is often advised for monitoring resistance training adaptations in non-ballistic exercises (e.g., squats, bench press) [64–66]. Considering these findings, accelerometers may best be used for the provision of feedback to enhance motivation and competitiveness during ballistic, high-velocity exercises [67]. However, accelerometers should not be used to track changes in performance (e.g., assessment of velocity against a fixed absolute load) nor to prescribe the loads or the volume of training sets when using velocity loss thresholds.

Of the studies that involved devices that were not accelerometer or linear transducers and assessed validity (Tables 6, 7), only three utilised a true gold-standard criterion [22, 24, 68]. When compared against a high-speed 3D motion-capture set-up, the Velowin opto-electronic device has demonstrated acceptable validity for both mean and peak velocity (CV = 6.5–7.5%); however, proportional bias in peak velocity may be present [68]. The optic laser Flex device has demonstrated acceptable validity for mean velocity during both free-weight squat and bench-press exercises across a range of loads (i.e., 20–100% 1RM) [22]. While there are small increases in variability at the lightest loads (i.e., 20% 1RM), between 40 and 100% of 1RM the typical error is approximately 0.02 m·s^−1^. It should be noted, though, that currently only mean velocity has been validated for the Flex, and other variables (e.g., peak velocity) still need to be compared against a gold-standard measure as these outputs may be most relevant to the lightest loads (e.g., 20% 1RM). Finally, with the increasing interest in monitoring resistance training performance, mobile phone apps have also become available [10, 24, 36, 69, 70]. While there is conflicting evidence [24, 36, 51, 70], it appears that substantial bias and error can be introduced when different devices and/or users implement these measuring tools [23]. Thus, practitioners should ensure thorough familiarisation and standardised protocols when using these applications.

### Reliability

A number of studies have investigated the reliability of linear transducers [10, 26, 36, 52] (Table 8). To date, the T-Force has had six separate studies investigate some aspect of reliability [10, 36, 54, 61, 71, 72]. Specifically, Courel-Ibañez et al. [36] have recently demonstrated the inter-device (i.e., two devices of the same manufacturer) reliability of mean, mean propulsive, and peak velocity, and shown the extremely low error (e.g., mean velocity CV = 1.0–2.1%) when completing the Smith machine bench press, squat, and the prone row. With respect to the intra-device (i.e., the same unit assessed across multiple repetitions), the study by Courel-Ibañez [36] demonstrated a slightly greater, but still relatively small, level of variability (i.e., mean velocity CV = 1.9–3.0%) within the same exercises. Furthermore, the authors presented findings to suggest that the Chronojump LPT exhibited slightly increased inter-device and intra-device error than the T-Force, with mean velocity variability ranging from 3.3 to 4.7% and 3.9 to 5.2%, respectively [36]. It should be acknowledged that the slightly greater intra-device variability values reported in this review may be due to the introduction of biological variation across repetitions (i.e., the ability of a human to perfectly replicate two repetitions with the exact same physical output). Furthermore, it is reasonable to suggest that these reliability outcomes may be negatively impacted when exercises are taken outside of a 2D plane (i.e., a Smith machine). During free-weight exercises, the within-device reliability of the GymAware PowerTool has been shown to be of a high standard [48, 52]. During the free-weight back squat, typical errors of 0.03–0.05 m·s^−1^ across loads of 20–90% of 1RM have been shown. However, this variability may be artificially inflated due to the time between testing occasions (i.e., 7 days) and the normal fluctuations in human performance [48]. Future research is still required to assess the inter-device reliability of this device.

While the accuracy of accelerometers during resistance training appears to be questionable, some accelerometer devices may have greater intra-device reproducibility [29] (Table 9). When placed on the bar, Push 2.0 has demonstrated acceptable reliability of both mean and peak velocity within the bench press at 60% and 90% of 1RM [29]. Furthermore, Hughes et al. [52] have shown conflicting reliability for this device during the Smith machine and free-weight squat, bench press, overhead press, and prone row when placed on either the bar or arm. Contrasting this, Beckham et al. [28] demonstrated that the Bar Sensei achieved both poor accuracy and poor reliability for mean and peak velocity measures during the free-weight barbell back squat. However, these values may have been inflated due to the period of time between testing occasions (i.e., 3–7 days) and the potential for biological variation to influence reliability outcomes [22]. Finally, the Beast Sensor has demonstrated extremely large variability (CV 24–55%) and systematic error at intensities of 45–85% of 1RM in a Smith machine back squat [10]. While a previous study has suggested that it demonstrates satisfactory reliability [69], the statistical approach has recently been questioned due to the pooling of repeated measures from a range of different intensities and consequently violating the assumption of independence [28]. Naturally, this may help to explain the contrasting results for this device and the high reliability correlation values previously reported [69].

Recent work by Perez-Castilla et al. [10] has compared seven commercially available devices in the Smith machine bench press across a range of loads (i.e., 45–85% of 1RM). Of these, the Speed4Lifts linear position transducer was found to demonstrate the greatest intra-device reliability (CV = 2.39–3.92%). This was closely followed by the Velowin, PowerLift, T-Force, and Chronojump that all demonstrated similar levels of reliability (CV =  ~ 3–6%) [10]. The authors reported that, outside of Speed4Lifts linear position transducer, all other devices demonstrated substantial heteroscedasticity when compared to a high-speed 3D motion-capture system. However, caution is required when interpreting these outcomes as the influence of between-day biological variation was not separated from the true technological error of the devices. Nevertheless, it should be noted that similar (CV =  ~ 4–8%) within-device reproducibility was observed for the Velowin and Powerlift when procedures were completed within-day [36] (Tables 10, 11). To the authors’ knowledge, the only study to separate these forms of variation when assessing within-device reliability is the recent work by Weakley et al. [22] on the optic laser FLEX device. This study investigated the reliability across a prolonged time (i.e., 21 days between testing occasions) with the use of a purposely designed calibrated rig. Mean velocity demonstrated an overall within-device typical error of ~ 4% with velocities ranging from 0.09 to 0.99 m·s^−1^. Additionally, this study demonstrated inter-device variance with both technological and biological variation accounted for. The authors concluded that the optic laser FLEX device exhibited acceptable inter-device reliability, suggesting that these devices can be used interchangeably (e.g., within a team environment where multiple barbells are set up). However, it should be noted that additional metrics (e.g., peak velocity) have recently been released by the manufacturers, and future research should be completed to assess these outputs.

While this review has considered a range of commercially available devices for the monitoring of resistance training, there are still several aspects that need further investigation. First, it should be acknowledged that the accuracy of these devices has been tested within a limited number of exercises (e.g., squat, bench press). Furthermore, a number of these studies have been done within a Smith machine which is expected to increase the reliability of the outputs. However, strength and conditioning practitioners often utilise a wide range of exercises and these are often done with free weights [73–76]. Additionally, some exercises that have greater horizontal displacement (e.g., Olympic weightlifting movements) have had minimal investigation. Therefore, future research is required for the validation of current technology using a wider range of exercises that include weightlifting movements and their derivates. Second, future research must consider the influence of biological variation when assessing the reliability of measurement devices. To date, all but one reliability study [22] have disregarded this consideration during within-device analysis, despite it being widely acknowledged that human performances fluctuate within-session and between-days. Thus, most of the within-device reliability research may mistakenly report reproducibility errors that are unrelated to the device. Finally, it is important to acknowledge the wide range of statistical approaches that have been used within the literature and that erroneous conclusions of validity and reliability may be drawn from an individual statistical value. For example, alone, correlations characterise the relationship between two outcomes, but they are incapable of describing any systematic bias that may exist. This has implications for concluding whether a device is truly accurate or reliable. Additionally, when interpreting error of individual devices, this should be put into context in relation to practical criteria or acceptable levels of disagreement. Thus, when quantifying the validity and reliability of these technologies, researchers are strongly advised to consider using a number of analyses that provide information about the level of agreement and the magnitude of errors that are associated with each device and compare these to appropriate criteria.

## Conclusions

The current review provides the reliability and validity of a range of different devices which are commercially available for the monitoring and prescription of resistance training. Generally, linear transducers have shown the greatest accuracy with mean concentric velocity the most assessed outcome. However, to date, only the GymAware [9, 25, 26, 54], T-Force [54], Open Barbell System [53], Tendo Fitrodyne [53, 54, 58], and Fitrodyne (Fitronic) [9] have been directly compared to a ‘true’ gold-standard 3D high-speed motion-capture system set-up during free-weight resistance training. When these devices have been directly compared to each other during free-weight exercises [9, 53, 54], it appears that the GymAware provides the greatest accuracy. This accuracy may be due to the device’s ability to account for horizontal displacement and variable rate sampling which is distinct to this device. Additionally, the T-Force demonstrates acceptable accuracy when exercise is performed within the Smith machine.

Accelerometer devices have shown promise, but the accuracy of these devices is still questionable [29, 37, 69]. Additionally, these devices are often validated against linear transducers which may introduce additional error that impacts the assessment of accuracy for the device [36, 38, 52, 61, 70]. Of the accelerometer devices, only the Push versions 1.0 and 2.0 [29, 37] and Beast Sensor [9] have been directly compared to a gold-standard criterion during free-weight exercises. Of these three devices, the Push 2.0 may have the greatest accuracy. However, it should be acknowledged that mean velocity from this device has been questioned [29], which limits its application to resistance training as this metric is widely recommended for use during non-ballistic exercises [12, 64, 65]. Finally, of the non-linear transducer and accelerometer devices, it appears that smart phone and tablet apps may be an alternative for a quick ‘snap-shot’ of training intensity, but substantial inter-device error may exist. Therefore, unless monitoring is done by a single individual with the same device, accurate tracking of performance may be limited [23, 36]. Nevertheless, the use of optic laser devices is a promising alternative that can provide accurate, real-time feedback [22]. While further research is still warranted on additional variables (e.g., peak velocity), this provides an additional cost-effective method for monitoring resistance training.

## Electronic supplementary material

Below is the link to the electronic supplementary material.

Supplementary file1 (DOCX 18 kb)

## Declarations

### Availability of data and materials

All data and materials reported in this systematic review are from peer-reviewed publications. All of the extracted data are included in the manuscript and supplementary files.
