# Supplementary material for: The Validity and Reliability of Commercially Available Resistance Training Monitoring Devices: A Systematic Review
Source: Sports Med. 2021 Jan 21;51(3):443–502. doi: 10.1007/s40279-020-01382-w (PMC7900050; doi:10.1007/s40279-020-01382-w)
Supplement: Supplementary file 1 — Supplementary file1 (DOCX 18 kb) [file 40279_2020_1382_MOESM1_ESM.docx]

| **Supplementary Table 1.** Modified Downs and Black methodological reporting quality questions | | |
| --- | --- | --- |
| **No.** | **Item** | **Score** |
| 1 | Is the hypothesis/aim/objective of the study clearly stated? | 0-1 |
| 2 | Are the outcome measures clearly stated? | 0-1 |
| 3 | Are details of the microtechnology (i.e. manufacturer, sampling frequency) stated? | 0-1 |
| 6 | Are the findings of the study clearly described? | 0-1 |
| 7 | Does the study provide estimates of the random variability in the data for the main outcomes? | 0-1 |
| 10 | Have actual statistical values been reported? | 0-1 |
| 16 | If any of the results were based on data dredging, was this made clear? | 0-1 |
| 18 | Were the statistical tests used appropriate? | 0-1 |
| 20 | Were the criterion measures used valid and reliable? | 0-1 |
|  | **Total** | **0-9** |
